# Prime editing links the split integrated stress response to pathogenic eIF2B mutations and white matter degeneration

**DOI:** 10.1038/s41419-025-08399-x

**Published:** 2025-12-27

**Authors:** Alessandra Scagliola, Annarita Miluzio, Martina Pauselli, Marcello Ceci, Stefano Biffo, Sara Ricciardi

**Affiliations:** 1 Department of Biological Sciences, DBS, University of Milan, Milan, Italy; 2 National Institute of Molecular Genetics, INGM, Romeo ed Enrica Invernizzi, Milan, Italy; 3 Department of Ecological and Biological Sciences, DEB, University of Tuscia, Viterbo, Italy

**Keywords:** Astrocyte, Demyelinating diseases

## Abstract

Vanishing White Matter Disease (VWMD) is a devastating, currently incurable neurodevelopmental disorder primarily affecting white matter. The prevailing view attributes VWMD to the activation of the canonical integrated stress response (c-ISR). However, recent studies have identified a novel, distinct pathway called the split ISR (s-ISR), though its activation has so far only been documented in mouse stem cells harboring a single eIF2B mutation, leaving uncertainty about whether it occurs in human cells, whether other mutations can trigger it, and what role it plays in the disease. Here, we used prime editing (PE) to engineer multiple eIF2B pathogenic mutations into HEK293T and induced pluripotent stem cells (iPSCs), generating human models. We demonstrated PE’s effectiveness and safety, marking the first successful application of PE for modeling VWMD. We found that all modeled mutations activate the s-ISR, indicating that this response is a common feature across VWMD mutations, and that it can be further amplified by stress-induced c-ISR and effectively suppressed by ISRIB. Mechanistically, we show that s-ISR hinders mutant iPSCs from achieving the high protein synthesis levels necessary for proper differentiation, expecially into astrocytes. This impairment disrupts their maturation process, directly linking s-ISR activation to the white matter abnormalities of VWMD.

## Introduction

Protein synthesis is a tightly controlled process essential for maintaining cellular homeostasis. Impairments in this process are linked to numerous inherited diseases [1].

Vanishing white matter disease (VWMD) is the first human hereditary disorder linked to defects in the initiation of protein synthesis as it results from mutations in any of the five non-identical subunits of the translation initiation factor eIF2B. This rare condition primarily affects white matter and manifests with progressive cerebellar ataxia, spasticity, and cognitive impairment, ultimately leading to neurological decline and death [2–6]. The age of onset varies widely, from early infancy to adulthood, with earlier onset correlating with a more rapid progression [6, 7].

VWMD remains an incurable condition, with current treatments only alleviating symptoms [4]. A major challenge in developing effective therapies is that the available human models do not adequately capture the complexity of the disease mechanisms influenced by various mutations. Therefore, developing models that more accurately replicate the disease’s pathophysiology remains highly a priority.

Genome editing has significantly advanced disease modeling and gene function studies [8–12]. Traditional CRISPR-Cas9 techniques, which depend on inducing double-strand DNA breaks and relying on homology-directed repair (HDR) for precise edits, however, face limitations such as low efficiency, a high rate of unintended insertions or deletions (indels), and dependence on nearby PAM sequences [13]. As a result, CRISPR technologies are continuously evolving, with new innovations that expand their capabilities and widen their range of applications [14].

These improvements given rise to various genome-editing tools, including programmable nucleases, base editors, and prime editors [14, 15]. Prime editing (PE) is particularly innovative because it combines the precise DNA recognition of CRISPR-Cas9 with a reverse transcriptase (RT) enzyme [15]. This allows it to introduce a wide range of genetic edits directly into the genome without causing double-strand breaks, greatly reducing the risk of unintended mutations at both on-target and off-target sites [15]. As a result, it overcomes many of the limitations associated with traditional CRISPR/Cas9 gene editing. Additionally, PE offers exceptional precision and versatility compared to other methods, making it an ideal tool for disease modeling [14, 16–18]. Despite its promising potential, however, PE has not yet been used to develop human models of VWMD.

eIF2B functions as the guanine exchange factor (GEF) for eIF2, positioning it as a critical regulatory node in controlling protein synthesis under a variety of cellular conditions [19, 20]. During cellular stress, eIF2 is phosphorylated at serine 51 on its α-subunit by several kinases, including PERK and GCN2 [21, 22]. This phosphorylation event inhibits eIF2B activity, resulting in a substantial decrease in global protein synthesis [21]. Importantly, heightened phosphorylation of eIF2α (P-eIF2α), together with reduced guanine nucleotide exchange activity of eIF2B, represents the hallmark of the ‘canonical’ integrated stress response (c-ISR). This pathway reprograms translation to facilitate cellular recovery. Beyond the suppression of overall protein synthesis, the ISR also induces the selective translation of specific mRNAs, notably the activating transcription factor 4 (ATF4), which plays a pivotal role in orchestrating cellular responses to stress by regulating gene expression [21, 23, 24].

VWMD is marked by a substantial decrease in eIF2B activity [25], which effectively mimics the effects of phosphorylated eIF2α. This makes it reasonable to suggest that VWMD involves a sustained activation of the canonical integrated stress response (c-ISR) [26–32].

However, recent studies have uncovered an alternative, distinct pathway known as the split ISR [33]. Similar to the c-ISR, the s-ISR is characterized by a decrease in protein synthesis rates, but, unlike the c-ISR, it depends on eIF4E-mediated translation induction of ATF4 without requiring the induction of phosphorylated eIF2α (P-eIF2α) [33]. The activation of the s-ISR in the context of VWMD has been primarily demonstrated in mouse embryonic stem (ES) cells harboring the R191H mutation in eIF2Bε [33]. To our knowledge, this approach has been limited to this single mutation in mice, and thus, our understanding of whether the s-ISR is also activated in human cells or if it represents a more general response triggered by other VWMD-associated mutations, as well as the implications of its activation for disease development and potential therapies, remains to be elucidated.

To explore this, we generated human VWMD models by introducing multiple pathogenic mutations in the eIF2B epsilon domain into HEK293T and iPS cells using PE and demonstrated its effectiveness and safety in engineering eIF2B5 variants, marking the first successful application of PE for modeling WVMD. Our findings show that all the modeled mutations activate the s-ISR in both cell types, indicating it is a common response across VWMD mutations. This pathway can be significantly amplified by stress-induced activation of the c-ISR and can be effectively rescued by ISRIB. Mechanistically, s-ISR prevents mutant iPSCs from achieving the high protein synthesis levels necessary for proper differentiation into astrocytes, disrupting their maturation and directly contributing to the white matter abnormalities seen in VWMD.

## Results

### Establishment of a PE strategy to model VWMD through targeted introduction of disease-causing eIF2B5 mutations

**Fig. 1:**
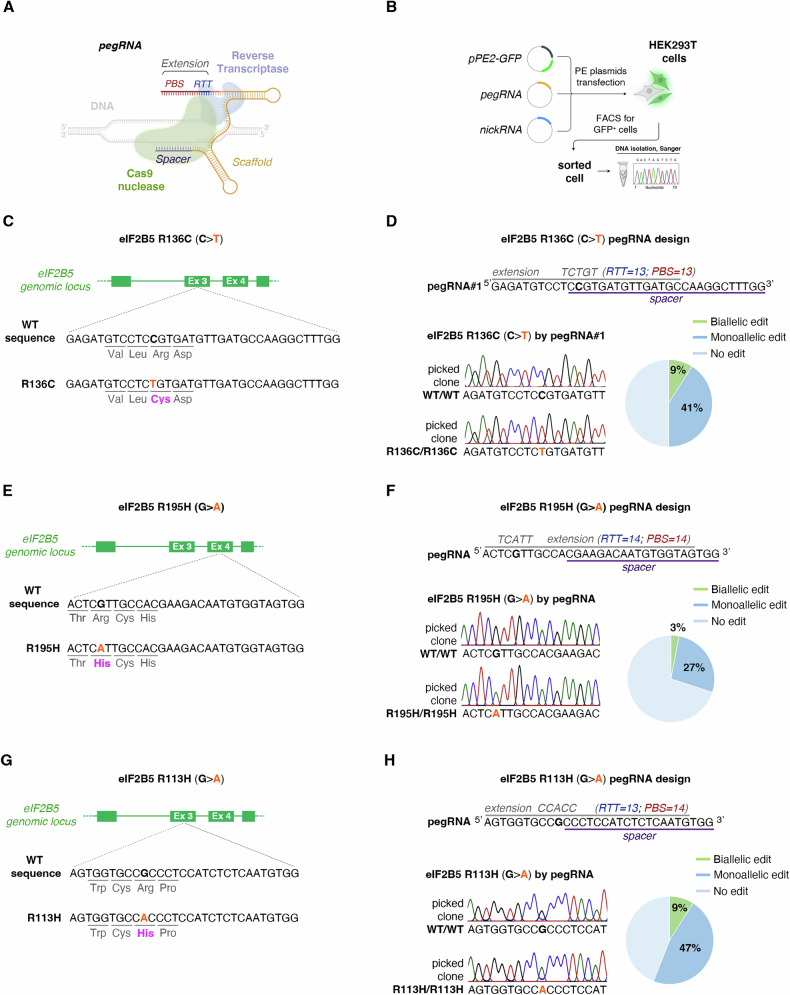
Prime editing enables precise and safe insertion of pathogenic mutations into the eIF2B5 gene in HEK293T cells. **A** Architecture of the pegRNA, which consists of a spacer (violet), scaffold (yellow), reverse transcriptase template (RTT, blue), and primer binding site (PBS, red). The prime editor protein is depicted in the background, with Cas9 shown in light green and the reverse transcriptase in purple. The target genomic DNA is in gray. PBS stands for the primer binding site, while RTT refers to the reverse transcriptase template. **B** Workflow overview for generating mutations in HEK293T cells using PE3. **C**, **D** pegRNA design for generating the εR136C mutation. The nicking sgRNA at +48 for pegRNA#1 is not depicted. Sanger validation of successful monoallelic edit, with editing efficiency shown to generate εR136C by pegRNA#1. **E**, **F** Design of the pegRNA to install the εR195H in HEK293T cells. Sanger validation confirms successful monoallelic edit with editing efficiency provided for the G → A substitution that generate the εR195H. **G**, **H** Design of the pegRNA utilized to install the εR113H mutation in HEK293T cells. Nicking sgRNA at +24 is not depicted. Sanger validation confirms successful monoallelic edit, with editing efficiency provided for the G → A substitution that generate the εR113H mutation.

To develop human models of VWMD that more accurately capture its underlying pathology, we introduced specific disease-causing mutations into both HEK293T and iPS cells using the PE system and following Anzalone et al. recommendations [15] (Fig. 1A, B). HEK293T cells served as a versatile platform for assessing the activation of the s-ISR, while iPSCs allowed us to study the implications of this activation for VWMD.

To begin with, we installed mutations in HEK293T cells chosen for their proven ability to express eIF2Bɛ and integrate it into functional eIF2B complexes [34].

Specifically, we installed the human c.406 C > T (p.Arg136Cys), in addition to c.584 G > A (p.Arg195His) and c.338 G > A (p.Arg113His), which will be referred to as εR136C, εR195H, and εR113H, respectively (Fig. 1C–H) [7, 25, 35]. Importantly, these disease alleles are located within the critical eIF2B epsilon catalytic domain, essential for its GEF activity. All variants are homozygous and associated with a spectrum of clinical severities, ranging from very severe to milder phenotypes. This diversity is expected to provide valuable insights into the effects of VWMD-causing mutations.

PE uses a nicking-Cas9 fused to a reverse transcriptase (RT) and a pegRNA, which contains the desired edit and a primer binding site (PBS) to initiate reverse transcription at the nicked strand, forming an edited DNA flap (PE2 system) (Fig. 1A) [15]. Efficiency is improved by creating a second nick with an additional sgRNA, called the PE3 system [15].

We developed a protocol for co-transfecting pegRNA and a plasmid containing the Cas9(H840A) nickase fused to an RT domain and GFP, known as the PE2 plasmid (Fig. 1B). The GFP allowed for selection by fluorescence-activated cell sorting (FACS) and clonal expansion of GFP-positive cells 24 h post-transfection (Fig. 1B and Supplementary Fig. S1A). Afterward, total genomic DNA was extracted, and the targeted region was sequenced (Fig. 1B).

Initially, we introduced the εR136C mutation, which is known to be associated with a severe form of VWMD (Fig. 1C). Following Anzalone et al. findings [15], which emphasize the crucial role of sgRNA effectiveness within the pegRNA for efficient PE, we employed CRISPOR sgRNAs efficiency predictor to guide the pegRNA design. The software identified sgRNA#1 and sgRNA#2 as the top candidates (Supplementary Table S1), which we then choose for further optimization.

pegRNA#1 included a 13-nucleotide PBS and a 13-nucleotide RT template (Fig. 1D), while pegRNA#2 featured a 13-nucleotide PBS and a 15-nucleotide RT template (Supplementary Fig. S1B). Given its higher editing efficiency compared to PE2 [15], we utilized the PE3 system throughout the study, and, therefore, during the evaluation of both pegRNA candidates, we co-delivered the PE3 nicking sgRNA.

Sequencing of cloned HEK293T cells confirmed biallelic insertion of the disease-causing mutation by both pegRNAs (Fig. 1D and Supplementary Fig. S1B). However, pegRNA#1 demonstrated significantly higher editing efficiency, achieving 50% on-target editing with 9% of cells exhibiting homozygous editing (Fig. 1D), compared to pegRNA#2, which showed 40% efficiency and only 3% homozygous editing (Supplementary Fig. S1B). We therefore selected pegRNA#1 for further experiments.

The rate of undesired mutations produced by genome editing tools is crucial for their therapeutic potential. We therefore assessed the specificity of PE by screening for off-target activity at the top three predicted sites from CRISPOR (Supplementary Fig. S1C) in the R136C mutant clones and unedited controls. Sequence analysis revealed no significant editing at these predicted off-target sites (Supplementary Fig. S1D), confirming that PE efficiently installs the homozygous εR136C mutation with base-pair precision while avoiding undesired modifications.

### PE precisely engineers a variety of eIF2B5 disease alleles

Next, we aimed to determine if the observed trends in PE efficiency apply to the other target sites. Continuing with the eIF2B5 locus, we utilized the PE3 system to insert the common point mutation εR195H (Fig. 1E), which is associated with a particularly severe form of VWMD [36]. Patients with this variant exhibit an early-infantile onset phenotype, averaging 5.3 months, and a progressive disease course resembling “Cree” leukoencephalopathy (CLE) in North American Indians. Notably, the developed animal models closely replicate key features of the human disease [29].

We designed a single pegRNA and a PE3-guide pair to introduce the εR195H mutation (Fig. 1F and Supplementary Fig. S2A). Sanger sequencing confirmed successful biallelic editing, although the efficiency was lower than for the εR136C mutation. Approximately 30% of the cells carried the desired mutation, with 3% showing homozygous edit (Fig. 1F). To improve editing efficiency, we optimized the pegRNA [15] by adjusting the PBS lengths and found that longer PBS lengths led to higher editing rates, with a 16 nt PBS being the most effective (Supplementary Fig. S2B).

Finally, we tested the effectiveness of PE in introducing the εR113H mutation (Fig. 1G), linked to the least severe form of the disease. We designed a pegRNA and PE3-guide pair, delivered using the same transfection protocol as for the εR136C and εR195H mutations (Supplementary Fig. S2C). Sanger sequencing revealed that 56% of the cells carried the desired edit, with 9% of these exhibiting homozygous conversion (Fig. 1H).

Collectively, these results demonstrated for the first time PE effectiveness and safety in precisely engineering pathogenic eIF2B5 variants, marking the first successful application of the PE technique to model VWMD. Furthermore, they also highlight the critical importance of testing various pegRNAs to ensure the achievement of the desired edits.

### The s-ISR is triggered by VWMD-relevant eIF2B5 variants

**Fig. 2:**
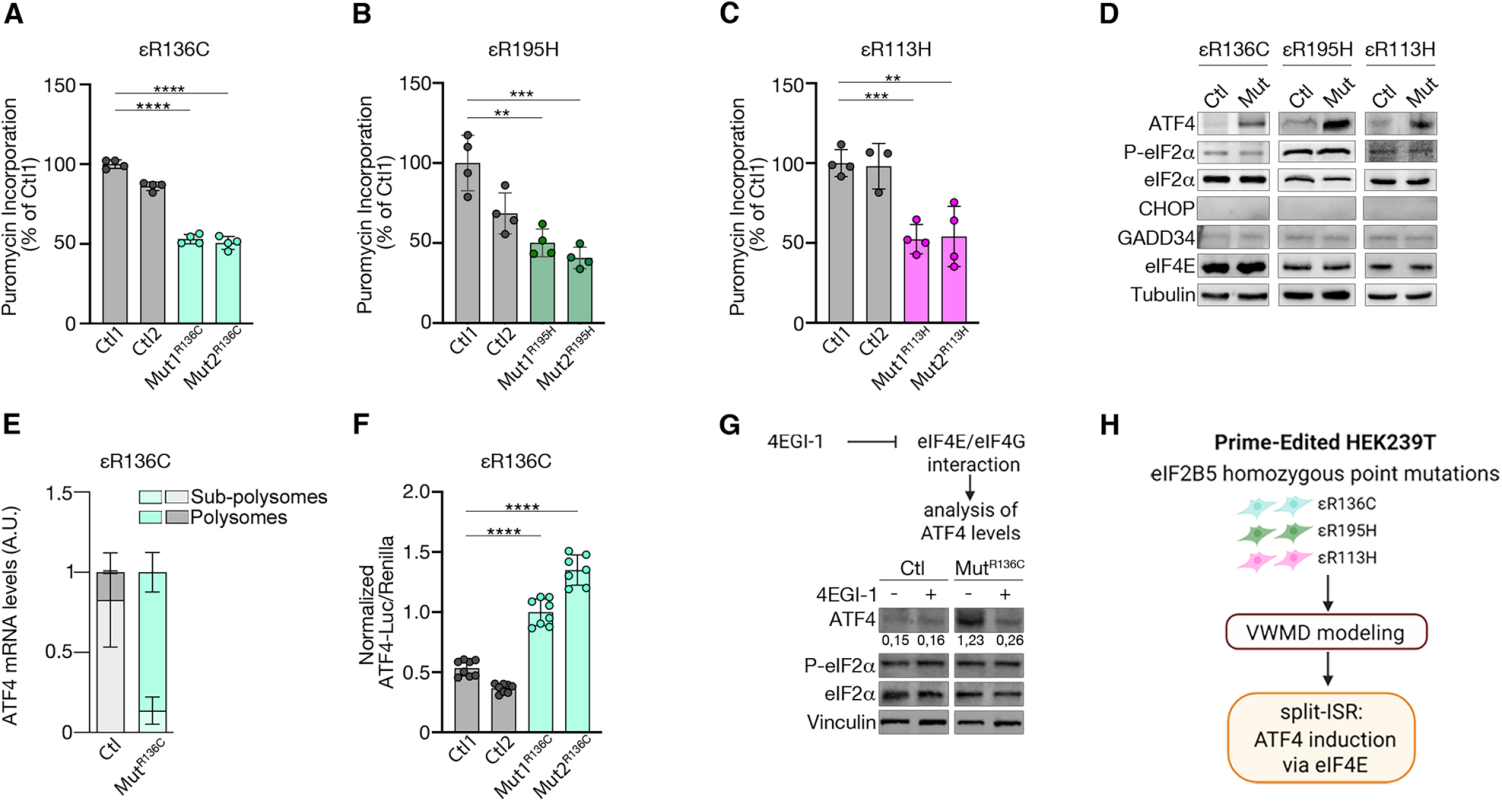
The activation of the s-ISR is a common feature observed across all eIF2B5 mutants generated via PE. Puromycin incorporation in Ctl and εR136C (**A**), εR195H (**B**) and εR113H (**C**) Mut cells. Data are the mean ± s.d. of the percentage relative to Ctl1. *p* values are determined by two-tailed Student’s *t* tests: **P < 0.01, ***P < 0.001, ****P < 0.0001. **D** Immunoblot representative of two independent experiments showing ATF4, P-eIF2α, eIF2α, eIF4E, CHOP, and GADD34 protein levels in Ctl and Mut cells. Tubulin was used as a loading control. **E** Quantification of ATF4 mRNA levels on polysomes and sub-polysomes reveals an increase in polysome-associated ATF4 transcripts in εR136C mutant cells. The data refer to the polysomes profile in Fig. S3A. Data are presented as the mean ± s.d. **F** Reporter analysis of ATF4 translational control. Luc reinitiation; Renilla cap dependent initiation. *p* values are determined by the unpaired t-test: ****P < 0.0001. **G** Representative immunoblot of two independent experiments showing disruption of the s-ISR-dependent induction of ATF4 with the eIF4E inhibitor 4EGI-1 in εR136C mutants. Vinculin was used as a loading control. Densitometric analysis of ATF4 levels are also indicated. **H** Schematic showing that s-ISR induction followed by eIF4E-dependent ATF4 translational control represents a common response across prime-edited VWMD mutations.

With our models established, we set out to determine whether the PE-installed eIF2B5 mutations activate the s-ISR. Polysome profiling revealed a translation initiation defect in εR136C cells, evidenced by a decrease in polysomes and an increase in the 80S monosome fraction (Supplementary Fig. S3A). This result was further supported by puromycin incorporation assay, which showed approximately a 40% decrease in puromycylation in εR136C cells, likely contributing to the slower growth observed in these cells (Fig. 2A and Supplementary Fig. S3B).

Similar to εR136C cells, both εR113H and εR195H mutants showed reduced level of global protein synthesis, though the reduction is less pronounced in εR195H cells (Fig. 2B, C). Additionally, in all three mutants, εR136C, εR113H, and εR195H, levels of ATF4 are increased while GADD34 and CHOP remained unchanged, and there was no evidence of eIF2α phosphorylation (Fig. 2D).

Polysome profiling combined with qRT-PCR revealed that, compared to only 20% in controls, approximately 80% of ATF4 mRNA is translationally activated in εR136C mutants (Fig. 2E), which was consistent with the increase in the corresponding protein levels. A luciferase reporter assay confirmed translational induction of the Atf4 mRNA, with εR136C mutants showing over a twofold increase in luciferase activity (Fig. 2F).

Further experiments demonstrated that blocking eIF4E with the 4EGI-1 abolished ATF4 induction in εR136C mutants (Fig. 2G), with similar results seen in εR113H and εR195H mutants (Supplementary Fig. S3C). Disrupting mTOR signaling with PP242 also prevented ATF4 induction in εR136C cells (Supplementary Fig. S3D), conclusively demonstrating that eIF4E is essential for ATF4 activation when eIF2B function is abrogated (Fig. 2H).

Overall, these findings support the P-eIF2α-independent s-ISR engagement in our VWMD models.

### eIF2B5 mutants activate the s-ISR worsened by episodes of ER stress that trigger the c-ISR, rescued by ISRIB

We then examined whether episodes of ER stress that activate the c-ISR could worsen the s-ISR. This idea aligns with clinical observations of VWMD, where disease progression often accelerates in response to stress, and with the therapeutic benefits seen from inhibiting the c-ISR using the eIF2B activator ISRIB, a small molecule that binds to and stabilizes the eIF2B complex in its active form [37, 38].

**Fig. 3:**
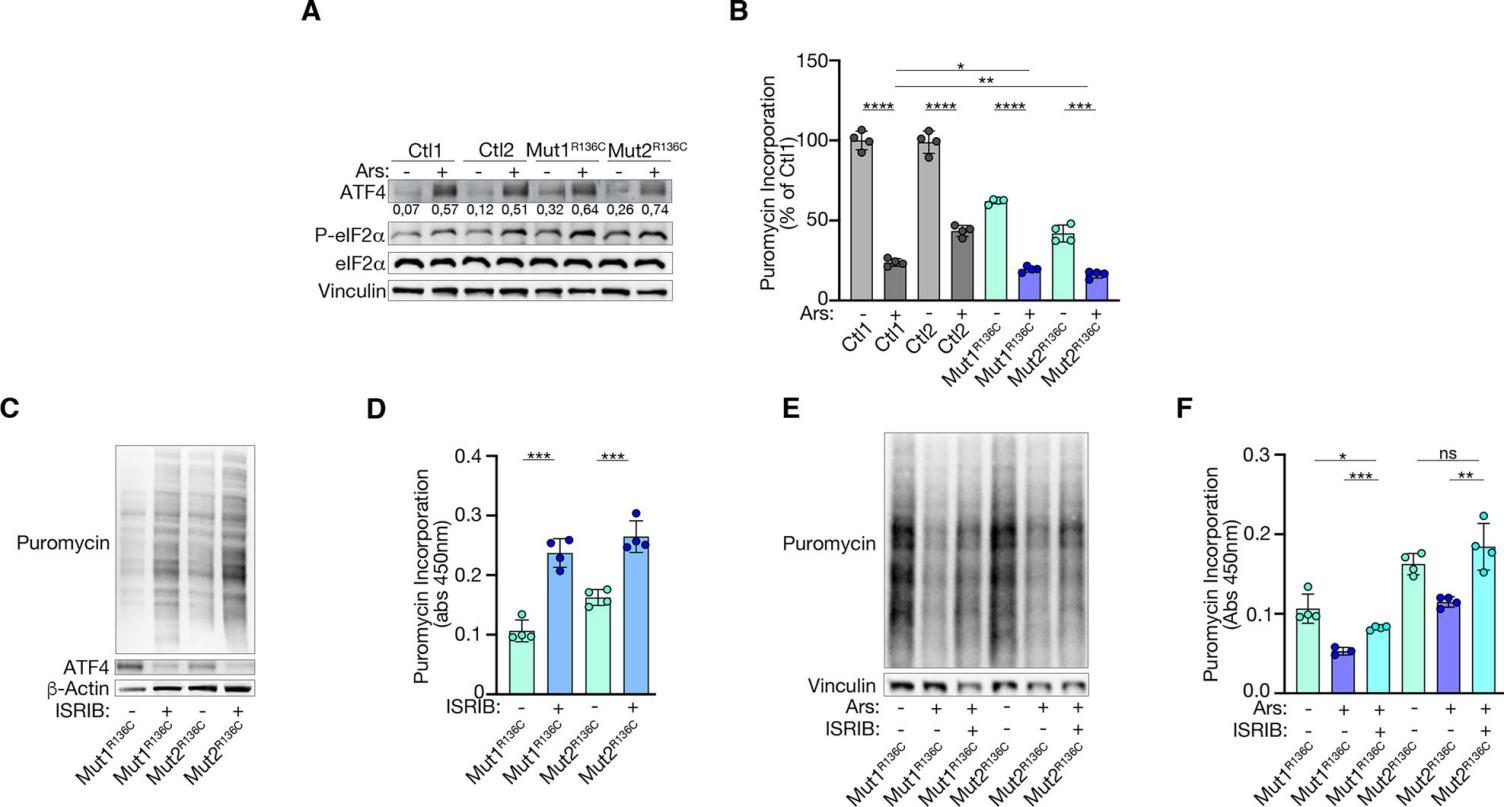
Activation of the c-ISR worsens s-ISR in εR136C mutant cells, rescued upon ISRIB treatment. **A** Representative immunoblots of two independent experiments showing ATF4, P-eIF2α, and eIF2α protein levels in Ctrl and εR136C Mut cells, left untreated or treated with Arsenite. Vinculin was used as a loading control. Densitometric analysis of ATF4 levels is also indicated. **B** Puromycin incorporation in Ctl and R136C Mut cells left untreated or treated with Arsenite. Data are the mean ± s.d. of the percentage relative to the untreated Ctl1. *p* values are determined by two-tailed Student’s *t* tests. *P < 0.05; **P < 0.01; ***P < 0.001; ****P < 0.0001. **C** Immunoblot representative of two independent experiments showing puromycin incorporation and ATF4 protein level in εR136C Mut cells, untreated or treated with ISRIB. β-Actin was used as a loading control. **D** Puromycin incorporation in εR136C Mut cells untreated or treated with ISRIB. Data are the mean ± s.d. of puromycin incorporation of treated and untreated samples. *p* values are determined by two-tailed Student’s *t* tests. ***P < 0.001. **E** Immunoblot representative of two independent experiments showing puromycin incorporation in εR136C Mut cells left untreated, treated with Arsenite or treated with both Arsenite and ISRIB. Vinculin was used as a loading control. **F** Quantitation of puromycin incorporation in εR136C Mut cells. Data are the mean ± s.d. of puromycin incorporation of treated and untreated samples. *p* values are determined by two-tailed Student’s *t* tests: ns not significant; *P < 0.05; **P < 0.01; ***P < 0.001.

To investigate this further, we treated cells with either the oxidative stressor arsenite (Ars) or thapsigargin (Tg), both of which are potent inducer of ER stress [33, 39, 40]. Compared to controls, arsenite further increased ATF4 levels across all three mutants (Fig. 3A and Supplementary Fig. S4A) while worsening translation defects in εR136C and εR113H (Fig. 3B and Supplementary Fig. S4B). Similarly, Tg treatment increased ATF4 levels in εR136C mutants (Supplementary Fig. S4C). Importantly, a hallmark of ER stress, increased P-eIF2α, was consistently observed following both Ars and Tg treatment (Fig. 3A and Supplementary Fig. S4C, D). These results indicate that both stressors activate the P-eIF2α-dependent c-ISR in our models, thereby amplifying the s-ISR response.

We next conducted a proof-of-concept study to explore the potential benefits of ISRIB [37, 41].

The addition of ISRIB markedly increased protein synthesis in both εR136C (Fig. 3C, D) and εR113H mutants (Supplementary Fig. S4D) while also preventing ATF4 induction in εR136C cells (Fig. 3C). Additionally, ISRIB nearly completely restored translation levels in εR136C cells exposed to arsenite (Fig. 3E, F), demonstrating its effectiveness in inhibiting the c-ISR even in the presence of the s-ISR.

Overall, these findings, for the first time, demonstrate that VWMD eIF2B5 mutants activate the s-ISR while still allowing cells to phosphorylate eIF2α, which then triggers the c-ISR and amplifies the s-ISR. Moreover, they highlight the potential of the eIF2B activator ISRIB to counteract these stress responses.

### The εR136C VWMD-relevant eIF2B mutation triggers the s-ISR in iPS cells

Next, we attempted to install the VWMD-relevant eIF2B εR136C mutation into iPS cells, an essential step to directly investigate the implications and the pathological significance of the s-ISR in VWMD.

**Fig. 4:**
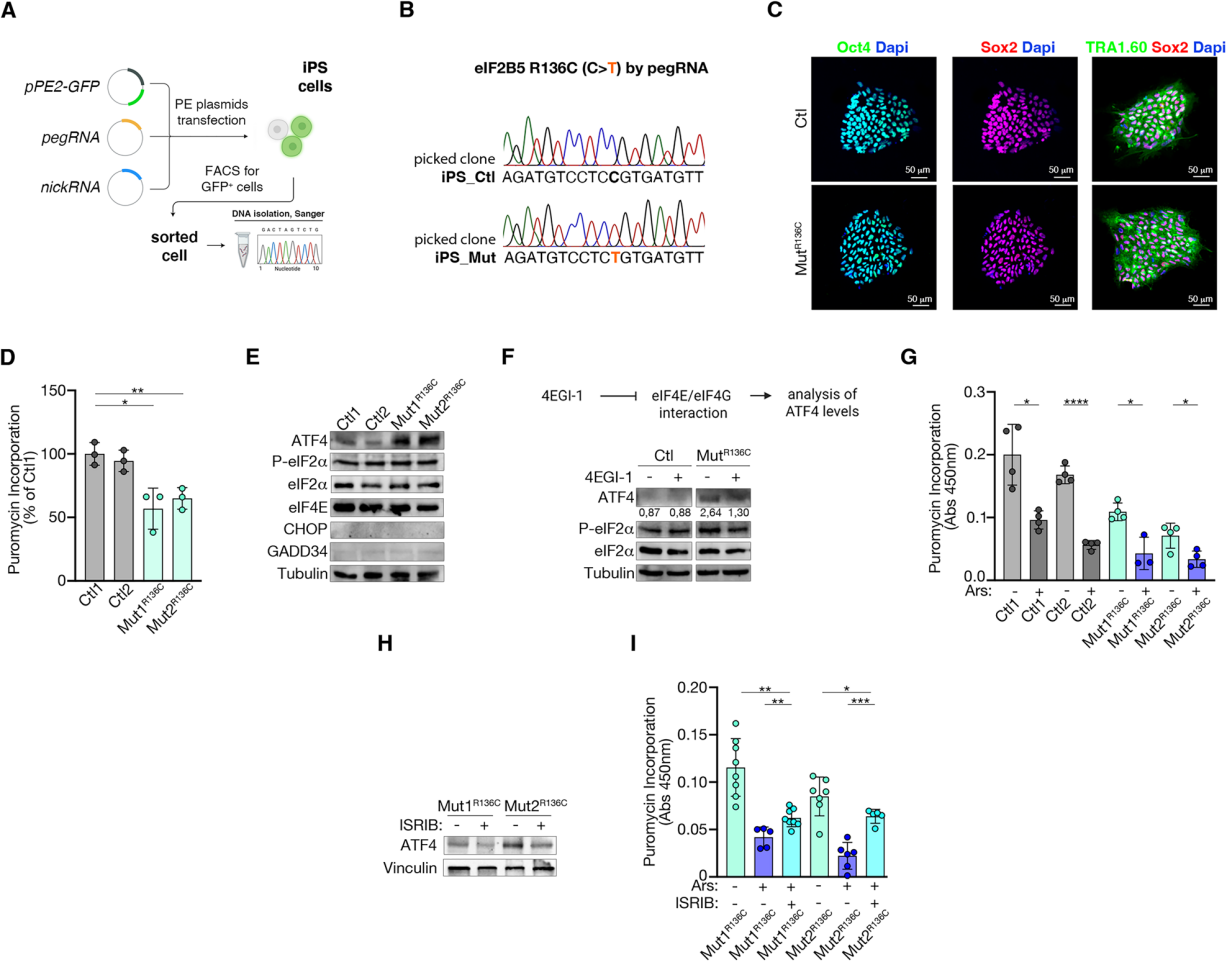
s-ISR is induced by the εR136C mutation in iPS cells. **A** Workflow overview for generating εR136C iPSCs. **B** Sanger validation of successful monoallelic edit, with editing efficiency shown for C → T substitution introduced by pegRNA#1. **C** Representative immunofluorescence images of Ctl and Mut iPSCs expressing the pluripotency markers OCT4, SOX2, and TRA-1-60. DAPI was used to stain nuclei. Scale bar, 50 μm. **D** Puromycin incorporation in Ctl and Mut iPSCs. Data are the mean ± s.d. of the percentage relative to Ctl1. *p* values are determined by two-tailed Student’s *t* tests: *P < 0.05; **P < 0.01. **E** Immunoblot representative of two independent experiments showing ATF4, P-eIF2α, eIF2α, eIF4E, CHOP, and GADD34 protein levels in Ctl and R136C Mut iPS cells. Tubulin was used as a loading control. **F** Immunoblot representative of two independent experiments showing that the eIF4E inhibitor 4EGI-1 disrupts the s-ISR-dependent induction of ATF4 in εR136C Mut iPS cells. Tubulin was used as a loading control. **G** Puromycin incorporation in Ctl and Mut iPSC lines left untreated or treated with Arsenite. Data are the mean ± s.d. *p* values are determined by two-tailed Student’s *t* tests. *P < 0.05; ****P < 0.0001. **H** Immunoblot representative of two independent experiments showing ATF4 protein level in εR136C Mut cells treated or left untreated with ISRIB. Vinculin was used as a loading control. **I** Puromycin incorporation in mutant iPSCs left untreated, treated with Arsenite or a combination of Arsenite and ISRIB. Data are the mean ± s.d. of puromycin incorporation of treated and untreated samples. *p* values are determined by two-tailed Student’s *t* tests: ns not significant; *P < 0.05, **P < 0.01; ***P < 0.001.

For the introduction of the mutation via PE, we selected the pegRNA#1, which showed the highest efficiency in HEK293T cells. We developed a co-transfection protocol with Lipofectamine Stem Transfection Reagent, followed by FACS and clonal expansion of transfected cells (Fig. 4A and Supplementary Fig. S5A). To enhance PE performance in iPSCs, we refined our approach by replacing the CMV promoter with the PGK promoter in the PE2 plasmid.

Direct sequencing of the PCR products generated around the targeted region revealed that approximately 20% of the PE cells exhibited the intended edit, with 2% showing homozygous editing (Fig. 4B and Supplementary Fig. S5B). These results indicate that PE was successful in iPS cells, although with somewhat lower efficiency compared to HEK293T cells.

The generated iPSCs carrying the εR136C mutation along with the mutation-free lines, were all validated for pluripotency using immunofluorescence (Fig. 4C).

We further confirmed the activation of the s-ISR in εR136C iPSCs, providing strong evidence that this stress pathway is indeed engaged. Mutant cells showed reduced protein synthesis (Fig. 4D and Supplementary Fig. S5C) and increased ATF4 levels, but not GADD34, CHOP, or P-eIF2α (Fig. 4E). Importantly, treatment with the eIF4E inhibitor 4EGI-1 abolished ATF4 induction in these cells (Fig. 4F).

Furthermore, similar to our findings in HEK293T cells, arsenite treatment worsened εR136C translation defects (Fig. 4G). We also evaluated the effects of ISRIB and found that it mitigates the c-ISR, restoring protein synthesis both under basal and stress conditions, while also preventing ATF4 induction in εR136C cells (Fig. 4H, I).

These results demonstrate that the εR136C mutation activates the s-ISR in iPSCs, thus establishing our iPSC model as a powerful tool to further investigate the role of s-ISR in VWMD.

### The s-ISR program triggered by the εR136C mutation prevents iPSCs from entering an elevated translation state that hinders their maturation into astrocytes

Stem cells generally keep protein synthesis low to remain undifferentiated, but they increase synthesis upon receiving differentiation signals [42]. Neural differentiation is typically triggered by inhibiting dual SMAD signaling with small molecules [43].

**Fig. 5:**
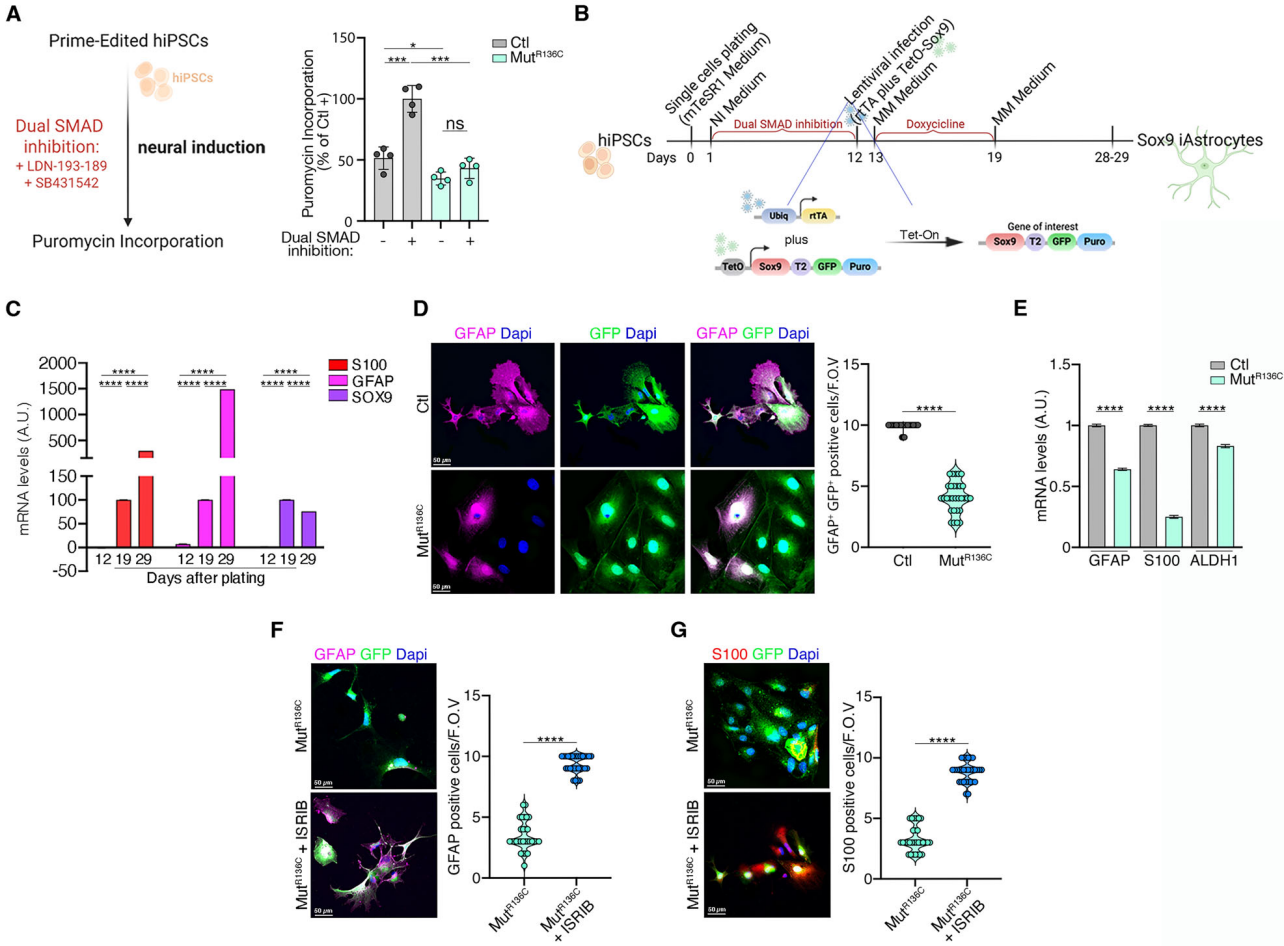
The εR136C-induced activation of the s-ISR prevents iPSCs from transitioning to an active translation state, thereby impairing their full development into mature astrocytes. **A** Left, workflow overview for neural induction by the dual SMAD inhibition method. Right, puromycin incorporation in control and mutant iPSCs following 1h treatment. Data are the mean ± s.d. of the percentage relative to the treated control. *p* values are determined by two-tailed Student’s *t* tests: *P < 0.05; ***P < 0.001. **B** Timeline of differentiation protocol for iPSC-derived astrocytes. NI neural induction medium. Lentiviral vector construct used to induce iPSCs differentiation into astrocytes is shown. Ubiq human Ubiquitin constitutive promoter, rtTA TET transactivator promoter gene, Sox9 Sox9 gene, Puro Puromycin resistance gene. **C** Representative qRT-PCR of two independent experiments of GFAP, S100B and SOX9 transcripts levels at different time points throughout the differentiation protocol compared to neural precursor cells (NPC). Values were normalized to 18S and expressed as percentage of the levels. *p* values are determined by two-tailed Student’s *t* tests: ****P < 0.0001. **D** Left, representative immunofluorescence images of GFAP (magenta) expression in Ctl and Mut iSOX9-astrocytes at 5 days after stopping doxycycline treatment. DAPI was used to stain nuclei. Scale bar, 50 μm. Right, quantification of the percentage of GFP^+^GFAP^+^ cells. Data are shown as the mean ± s.d. *p* values are determined by two-tailed Student’s *t* tests: ****P < 0.0001. **E** Representative qRT-PCR of two independent experiments showing relative mRNA levels (Mut *vs* Ctl) of GFAP, S100B, and ALDH1L1 transcripts in Ctl and Mut iSOX9-astrocytes at 5 days after stopping doxycycline treatment. Values were normalized to 18S and data are the mean ± sd. *p* values are determined by two-tailed Student’s *t* tests: ****P < 0.0001. **F** Left, representative immunofluorescence images showing GFAP (magenta) expression in Ctrl and Mut iSox9-GFP astrocytes, either untreated or treated with ISRIB. DAPI was used to stain nuclei. Scale bars, 50 μm. Right, quantification of the percentage of GFP^+^GFAP^+^ cells. Data are shown as the mean ± s.d. *p* values are determined by two-tailed Student’s *t* tests: ****P < 0.0001. **G** Left: representative immunofluorescence images showing S100 (red) expression in Ctrl and Mut iSox9-GFP astrocytes, either untreated or treated with ISRIB. DAPI was used to stain nuclei. Scale bars, 50 μm. Right, quantification of the percentage of GFP^+^S100^+^ cells. Data are shown as the mean ± s.d. *p* values are determined by two-tailed Student’s *t* tests: ****P < 0.0001.

We treated both control and mutant iPSCs with these molecules and sampled after 1 h to measure translation via puromycin incorporation (Fig. 5A). Notably, control cells displayed a significant translational burst, while mutant iPSCs failed to respond (Fig. 5A). This prompted us to investigate whether these translational defects alter mutant cells differentiation. Since astrocytes are most affected in VWMD [28, 29, 31, 32], we used the newly generated εR136C iPSCs to specifically guide their differentiation toward the astrocyte lineage (Fig. 5B).

We employed a rapid and efficient differentiation method, much shorter than existing protocols [44]. We obtained pure, mature astrocytes by inducing SOX9 overexpression in iPSC-derived neural progenitor cells (NPCs) for six days following 12 days of dual-SMAD inhibition (Fig. 5B). qRT-PCR verified persistent SOX9 expression, which decreased after doxycycline removal.

Six days after induction, GFAP and S100B transcripts were readily detectable (Fig. 5C), and, in line with these results, most GFP^+^ iSOX9 astrocytes expressed both markers (Fig. 5D and Supplementary Fig. S6), confirming successful differentiation.

We next compared mutant and control iAs 5 days after doxycycline withdrawal. Immunostaining for GFAP and S100B showed a reduced number of GFP^+^SOX9^+^ cells in mutant cells (Fig. 5D and Supplementary Fig. S6), indicating impaired astrocyte maturation. These findings were further supported by quantitative RT-PCR, which showed significantly lower levels of GFAP, S100B, and ALDH1L1 transcripts in mutant cells (Fig. 5E).

Importantly, the maturation impairment observed in mutant iPSC-derived astrocytes was successfully rescued by differentiating them in the presence of ISRIB, which restored GFAP- and S100B-positive SOX9 astrocytes to levels comparable to controls (Fig. 5F, G).

This finding links s-ISR activation to the white matter abnormalities characteristic of VWMD, providing important insights into the potential contribution of the s-ISR pathway to disease development.

Overall, our data support a model wherein the s-ISR program prevent iPSCs from achieving the high translational levels necessary for proper differentiation, thereby impairing their development into astrocytes. This mechanism could be the first to explain the white matter cell defects characteristic of VWMD.

## Discussion

A thorough understanding of gene function and variants is crucial for targeted treatments, but the scarcity of accurate human models for VWMD hampers progress [45–47]. Therefore, creating human models that more closely mirror the disease’s true pathophysiology remains highly a priority.

In this study, we established a PE strategy to generate human model of VWMD by engineering multiple pathogenic eIF2B5 mutations and demonstrated its high flexibility, efficiency and safety across various cell types, including iPSCs, through a single-step process in engineering eIF2B5 variants, marking the first successful application of PE for modeling WVMD.

Genotyping of iPSC clones revealed, however, slightly lower editing efficiency compared to HEK293T cells, likely due to the difficulty of delivering multiple plasmids, such as the prime editor, pegRNA, and nicking sgRNA, simultaneously into iPSCs.

Advanced systems like PE4 and PE5 have improved PE in iPSCs by disrupting the DNA mismatch repair (MMR) pathway with a dominant negative MLH1 (MLH1dn), combined with PE2 and PE3 [48–50]. Their efficiency is further boosted when paired with PEmax, an optimized and more efficient prime editor.

These technological advancements, along with more recent developments [51], strengthen our confidence that, with further optimization, we will be able to reliably generate robust and expandable VWMD-iPSC models. Such models will enhance our understanding of the mechanisms driven by the diverse mutations associated with VWMD and, ultimately, speed up the development of therapies.

The efficiency of PE is strongly influenced by the design of the pegRNA [15]. In our hands, we observed that pegRNA design has a significant impact on editing success, with optimal PBS and RT template parameters varying depending on the target site.

The fidelity of genome editing tools is crucial for their therapeutic potential. To this end, we evaluated PE’s specificity by screening for off-target effects. Our results showed no detectable off-target mutations at sites resembling the target region. Although limited by sample size, this absence of genome-wide off-target activity is highly encouraging for the continued development of PE-based therapeutics.

Our findings demonstrate that s-ISR is a more common and widespread response, observed across all the VWMD models we developed. This challenges the traditional view that sustained activation of the c-ISR is the primary driver of VWMD. Notably, the s-ISR is activated under mild stress conditions, whereas severe stress tends to shift the response toward the c-ISR. All the mutations we modeled with PE triggered the s-ISR, which can be intensified by ER stress-induced c-ISR activation and reduced by ISRIB. These findings may partly explain why VWMD worsens under stress and support the therapeutic potential of the c-ISR inhibition with ISRIB.

Our study also shed light on the potential role of the s-ISR in VWMD development, potentially explaining the white matter defects characteristic of the disease. However, further studies are needed to confirm whether similar issues occur with other mutations, using different differentiation methods, and to evaluate the long-term effects.

In summary, we demonstrated the potential of PE to precisely and safely generate human models of VWMD. We also reveal that the activation of s-ISR pathway is a common mechanism underlying VWMD mutations and plays a role in disease development. Actually, the activation of the s-ISR prevents iPSCs from transitioning to an active translation state, thereby impairing their full development into mature astrocytes. These insights deepen our understanding of the molecular and cellular basis of VWMD, a rare, incurable disorder with significant unmet medical needs.

## Materials and methods

### Mammalian cell culture conditions

HEK293T cells were cultured in Dulbecco’s modified Eagle medium with GlutaMax (Thermo Fisher Scientific) supplemented with 10% fetal bovine serum (Thermo Fisher Scientific) and 1× penicillin-streptomycin (Thermo Fisher Scientific) at 37 °C with 5% CO_2_.

### Culture and maintenance of iPSCs

iPSCs were obtained from the European Bank of induced pluripotent Stem Cells (EBiSC) (depositor, Wellcome Sanger Institute; WTSIi004-A), verified pluripotent and contamination free.

iPSCs were cultured on human embryonic stem cell (HESC)-qualified Matrigel (Corning) coated plates in mTeSR1 (Stem Cell Technologies) with penicillin-streptomycin. At 80% confluence, cells were passaged with ReLeSR (Stem Cell Technologies), incubated for 3 min and replated at a 1:6 density.

### Drug treatments

Where indicated, cells were treated with either 40 µM Arsenite, 1 µM ISRIB for 1 h or a combination of 40 µM Arsenite and 1 µM ISRIB for 1 h.

Where indicated, cells were treated with 400 nM Thapsigargin for 1 h.

Where indicated, cells were treated with 200 µM 4EGi-1 (Tocris Bioscience) or 1 µM PP242 (Millipore) for 4 h.

For dual-SMAD inhibition, iPSCs were treated with 1 µM LDN-193,189 (Miltenyi) and 10 µM SB431542 (Tocris) for 1 h.

Where indicated, iSOX9-astrocytes were treated with 300 nM ISRIB, with daily medium refreshment and ISRIB addition.

### pegRNA design and construction of plasmids

pegFinder pegRNA designer for CRISPR PE (http://pegfinder.sidichenlab.org), was used to design PE and nicking guides [52]. eIF2B5 wild-type/reference and edited sequences were used as input.

Cloning of pegRNA plasmids was conducted according to previously described protocols. Briefly, the pU6-pegRNA-GG-Vector (Addgene #132777) was digested with BsaI-HFv2 (NEB), and the 2.2 kb fragment was isolated. Oligonucleotide duplexes containing the desired pegRNA spacer, extension, and scaffold sequences were ordered with the appropriate overhangs and annealed in T4 Ligation Buffer (NEB), along with T4 PNK to phosphorylate the oligos. The diluted (1:100) annealed duplexes were then ligated into the digested vector using Quick Ligase (NEB).

Similarly, to generate nicking sgRNA plasmids, the diluted (1:100) secondary nicking sgRNA duplexes were ligated into the BsmBI-digested U6 sgRNA vector backbone (the lentiGuide-Puro vector; Addgene #52963).

In our PE protocol for iPS cells, we replaced the CMV promoter with the PGK promoter in the PE2-GFP plasmid (Addgene #132776) to enhance expression efficiency.

Sequences for all pegRNAs and nicking sgRNAs are provided in Supplementary Tables S2 and S3.

### HEK293T cell transfection with PE plasmids

HEK293T cells (50,000/well) were seeded in 24-well plates (Corning). After 24 h, at 70–80% confluence, they were transfected with PE plasmids and 1 µl Lipofectamine 2000 in Opti-MEM (Thermo Fisher Scientific), following the manufacturer’s instructions. For transfections, the plasmids amount used were 375 ng PE, 125 ng pegRNA, and 40 ng sgRNA. Cells were cultured until FACS.

### iPSCs transfection with PE plasmids

iPSCs were dissociated with Accutase, seeded at ~1 × 10^5^ cells per well in Matrigel-coated 6-well plates with 10 µM ROCK inhibitor Y-27632 (bio-techne). After 24 h, they were transfected at 70–80% confluence with PE plasmids and 6 µl of Lipofectamine Stem Transfection Reagent (Thermo Fisher Scientific) in Opti-MEM, using 3 µg PE, 750 ng pegRNA, and 300 ng sgRNA for PE3. Cells were cultured until FACS.

### Preparation of HEK293T and iPS cells for fluorescence-activated cell sorting (FACS)

24 h after transfection, HEK293T cells were trypsinized using Trypsin (Gibco), washed twice with phosphate-buffered saline (PBS), and subjected to cell sorting.

Similarly, 24 h post-transfection, iPSCs were dissociated using Accutase (Sigma) for 5 min, washed twice with PBS, resuspended in PBS supplemented with 10 μM ROCK inhibitor and subjected to cell sorting.

Green fluorescent protein (GFP)-positive cells were isolated using a BD FACSAria III flow cytometer (BD Biosciences). Refer to Figs. S1, S2 and S5 for the FACS gating strategy.

After FACS, HEK293T cells were counted and plated at 0.5 cells/well in 96-well plates with 100 µl medium.

Sorted iPSCs were resuspended in mTeSR1 with antibiotics and ROCK inhibitor, then plated in 48-well plates. Cells were cultured for 10–14 days to generate monoclonal populations, which were expanded, genotyped, and sequenced as described below.

### Genomic DNA preparation and genotyping

Genomic DNA was extracted using the DNeasy Blood and Tissue Kit (QIAGEN) according to the manufacturer’s protocol. Regions of interest were amplified with Phusion High-Fidelity DNA Polymerase (Thermo Fisher Scientific) using 200 ng of gDNA, following the manufacturer’s instructions. Primers, listed in Supplementary Table S4, were synthesized by Eurofins Genomics.

The PCR products were purified using the QIAquick® Gel Extraction Kit (QIAGEN) in accordance with the manufacturer’s protocol and sent for Sanger sequencing (Eurofins Genomics). The results were analyzed using SnapGene (Dotmatics Limited, Boston, MA, USA, version 7.1.2).

### Off-target analysis

The potential off-target sites of pegRNA#1 were identified using the online tool: http://crispor.tefor.net. Briefly, we selected the top three most likely off-target hits for pegRNA#1 that fell within gene-coding regions. These sites were amplified by genomic PCR (primers are listed in Supplementary Table S4), and the resulting amplicons were subjected to Sanger sequencing to assess the absence of alterations.

### In vitro measurement of protein synthesis

For the measurement of protein synthesis, cells were treated with 5 μg/mL puromycin for 10 min. Puromycin incorporation was then assessed by western blotting, as described later, or by ELISA [53].

For the ELISA assay, cells were lysed, and equal protein amounts were loaded onto 96-well ELISA plates and blocked with 5% BSA for 1 h at room temperature. Plates were then incubated with anti-puromycin antibody for 1 h, washed, then treated with HRP-conjugated anti-mouse antibodies for 30 min. After washing, TMB substrate was added to detect peroxidase activity, and absorbance was measured using a Bio-Rad Model 680 Microplate Reader.

### Polysomal profiles

Polysome profiles were prepared from HEK293T cells as follows. Cells were lysed in 30 mM Tris-HCl, pH 7.5, 100 mM NaCl, 30 mM MgCl2, 0.1% NP-40, 100 mg/ml cycloheximide and 30 U/ml RNasin. Lysates were clarified at 12,000 × *g* for 10 min at 4 °C. Equal RNA amounts were loaded onto a 15–50% sucrose gradient and centrifuged at 4 °C in a SW41Ti Beckman rotor for 3 h 30 min at 39,000 r.p.m. Absorbance at 254 nm was recorded by BioLogic LP software (BioRad) and ten fractions (1 ml each) were collected for subsequent RNA extraction.

### SDS-PAGE and western blotting

SDS-PAGE and western blotting were performed on protein extracts obtained as previously described [54].

Chemiluminescent signals on the blots were detected using SuperSignal West Pico PLUS Chemiluminescent Substrate (Thermo Fisher Scientific) and images were acquired using the Chemidoc MP Imaging System (Bio-Rad). All primary antibodies are listed in Supplementary Table S5.

### RNA isolation and qPCR

Total RNA isolation was performed using TRIzol™ Reagent (Thermo Fisher Scientific) according to the manufacturer’s instructions. For total, subpolysomal and polysomal RNA extractions from sucrose gradient aliquots, samples were incubated with 100 μg/mL proteinase K and 1% SDS for 2 h at 37 °C. RNA was then extracted by phenol/chloroform-isoamyl alcohol procedure.

Reverse transcription was performed using SuperScript IV VILO Master Mix with ezDNase (Thermo Fisher Scientific). Quantitative PCR (q-PCR) was carried out with Platinum SYBR Green qPCR SuperMix-UDG with ROX (Thermo Fisher Scientific) on QuantStudio™ 3 Real-Time PCR System (Thermo Fisher Scientific). All primers used are listed in Supplementary Table S4. Experimental samples were analyzed and normalized with the expression level of housekeeping genes, 18S or Actin. Relative quantification was performed by applying the 2^−ΔΔCt^ method.

### Immunostaining and fluorescence microscopy

Immunofluorescence was performed as previously described [54]. Briefly, cells were fixed in 2% paraformaldehyde with 3% sucrose for 10 min, permeabilized with 0.5% Triton-X100 in 20 mM HEPES, pH 7.4, then blocked. After blocking, cells were treated with the following primary and secondary antibodies: mouse monoclonal anti-GFAP (2.2B10) (1:1000, Thermo Fisher Scientific), rabbit polyclonal anti-S100B (1:1000, Proteintech), Goat anti-Rabbit IgG (H + L) Alexa FluorTM 488 and Goat anti-Mouse IgG (H + L) Alexa FluorTM 647 secondary antibodies (Thermo Fisher Scientific). Nuclei were stained with DAPI, and images were acquired using a confocal microscope (Leica Microsystems, model SP5, equipped with 8 laser lines and 4 PMT detectors).

### Lentiviral constructs and virus production

The rtTA (reverse tetracycline-controlled transactivator) and tetO-Sox9-GFP lentiviral vectors were obtained from Addgene (#20342) and VectorBuilder (#VB220503-1035tqr), respectively. Lentiviruses were produced as follows: HEK293T cells were plated at a density of 125,000 cells/cm² in DMEM, high glucose, Glutamax (Thermo Fisher Scientific) supplemented with 10% FBS and 1× penicillin-streptomycin. The following day, when the cells reached 90% confluency, they were transduced with VSV-G, PMDL g/pRRE, and pREV plasmids, along with a plasmid encoding either M2rtTA or SOX9, using calcium phosphate transfection. After 24 h, the medium was replaced with regular HEK293T cell medium. Lentiviruses were harvested 48 h post-transfection, pelleted by centrifugation (20,000 × *g* for 120 min), resuspended in PBS, aliquoted, and frozen at −80 °C. Only virus preparations with >90% infection efficiency, as assessed by GFP expression, were used for the experiments.

### Generation of iSOX9-astrocyte from iPS cells

iSOX9-astrocyte differentiation was performed as previously described with some modifications [44]. Briefly, single-cell suspensions of Wtsli004-A iPSCs were plated at a density of 150,000 cells/cm² on Matrigel-coated plates in mTeSR1 medium supplemented with 1X Revitacell (Thermo Fisher Scientific). The following day, the medium was changed to neuroinduction (NI) medium: a 1:1 mixture of Neurobasal medium (Thermo Fisher Scientific) and DMEM/F12 supplemented with 0.5× Glutamax (Thermo Fisher Scientific), 50 U/ml penicillin-streptomycin, 0.5× B27 (Thermo Fisher Scientific), 0.5× N2 (Thermo Fisher Scientific), 0.5× MEM-NEAA (Thermo Fisher Scientific), 0.5× sodium pyruvate, 0.025% human insulin (Sigma), and 50 µM 2-mercaptoethanol (Thermo Fisher Scientific), 1 µM LDN-193,189 (Miltenyi) and 10 µM SB431542 (Tocris) and changed daily for 12 days.

On day 12, cells were detached with Accutase, suspended as single cells, and plated at 100,000 cells/cm² on Matrigel in NMM with 20 ng/ml bFGF (bio-techne) and 1X Revitacell (Thermo Fisher Scientific). The next day, Sox9 and m2rtTA lentiviral vectors were added. After 1 day, media was changed to maturation medium: a 1:1 mix of Neurobasal and DMEM/F12 with 1× N2, 1× sodium pyruvate, 1× Glutamax™, 0.5 mM N-acetylcysteine (Sigma), 0.1 mM dbcAMP (Sigma), 10 ng/ml CNTF (Peprotech), 10 ng/ml BMP4 (Peprotech), and 5 ng/ml HB-EGF (Peprotech).

For the first 6 days, the maturation medium was supplemented with 3 µg/ml doxycycline and replaced every other day. Subsequently, the medium was changed twice a week.

### Reporter assays

The human ATF4 uORF reporter (courtesy of Dr. R. Wek) [55] was used for dual luciferase assay, following Promega instructions after protein quantification, with results from three experiments expressed as the firefly/Renilla ratio (relative light units).

### Quantitation and statistical analysis

Statistical analyses were performed using GraphPad Prism (Version 8, GraphPad Software). The data are expressed as mean ± sd. or s.e.m. The number of independent replicates, error bars, *P* values, and statistical tests are reported in the corresponding figure legends. The following symbols are used in the figure legends for *P* values: n.s., not significant; *P < 0.05; **P < 0.01; ***P < 0.001; ****P < 0.0001.

Quantitation of the western blots and quantitation of the percentage of GFP-positive SOX9^+^ cells were performed using ImageJ analysis software.

## Supplementary information

Supplementary Information

Western-blot_full unedited gel

Supplementary_Table_S1-S5

## Source data

Source_data

## Data Availability

This study does not involve datasets requiring public deposition. All experimental procedures and results are thoroughly described within the manuscript and are accessible in the online Supplementary Data, Supplementary Material, and Source data files.
