# Supplementary material for: Prime editing links the split integrated stress response to pathogenic eIF2B mutations and white matter degeneration
Source: Cell Death Dis. 2025 Dec 27;17(1):141. doi: 10.1038/s41419-025-08399-x (PMC12848117; doi:10.1038/s41419-025-08399-x)
Supplement: Supplementary file 1 — Supplementary Information [file 41419_2025_8399_MOESM1_ESM.pdf]

Supplemental Materials for

Prime Editing Links the Split Integrated Stress Response to Pathogenic eIF2B Mutations and White Matter Degeneration

Scagliola et al.

*Corresponding author. Email: ricciardi@ingm.org

This PDF file includes:

Figs. S1 to S6 and Supplementary legends

Supplementary Figure 1

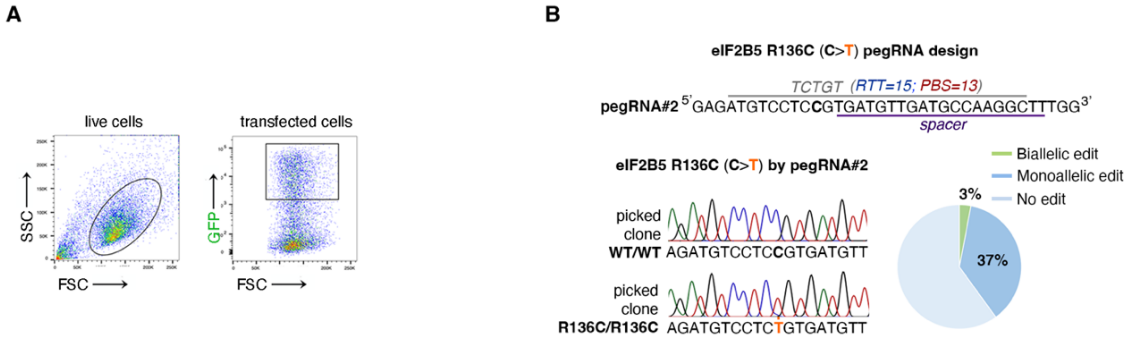

C

Potential pegRNA#1 off-target sites

Gene	Chr	Sequence (including mismatches)	No of Mismatches	Score
PIK3IP1	22	5'-CCTTAGCAGCAGCATCACTG *TGG*-3'	4	0.25
TACC2	10	5'-CCTGGCCAACAACATCACCG *TGG*-3'	4	0.17
F3	1	5'-CCTTGCCAAAAACATCCCGG *AGG*-3'	4	0.09

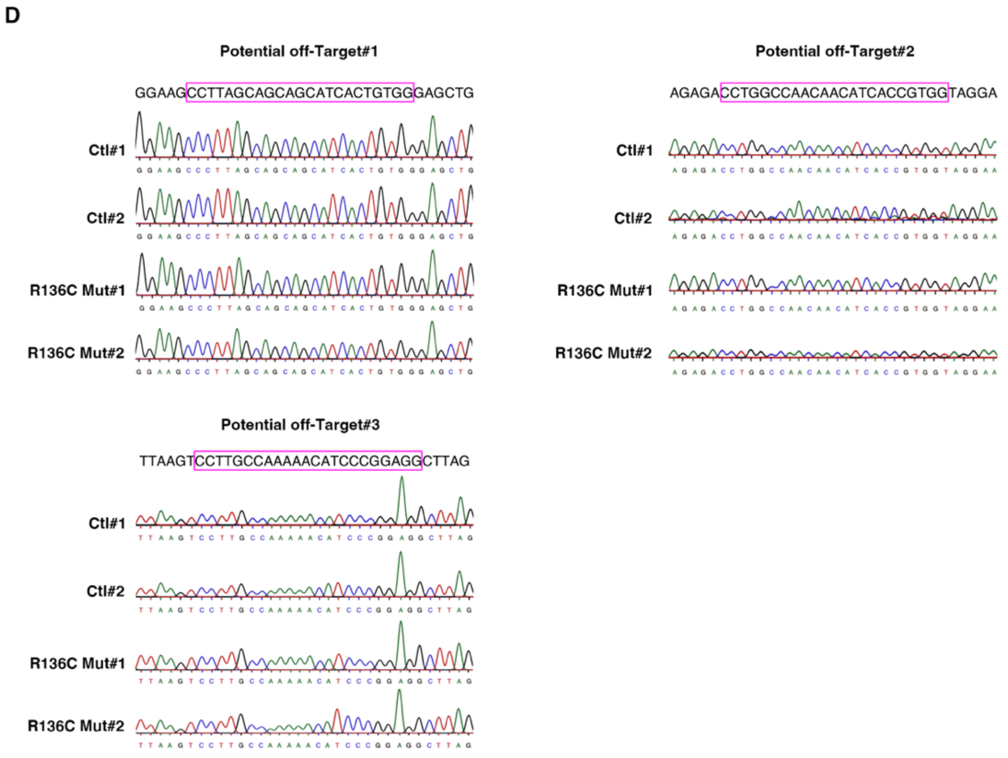

Supplementary Figure 1. Analysis of potential off-target site in HEK293T cells for PE-mediated generation of the εR136C mutation. (**A**) Representative FACS plot and gating strategy to sort GFP+ HEK293T cells. (**B**) Sanger validation of successful monoallelic edits, with editing efficiency shown for C → T substitution to generate εR136C by pegRNA#2. (**C**) The three most likely potential off-target genomic sites identified by CRISPOR, which fall within coding regions for pegRNA#1 designed to install the εR136C mutation, are shown. These three loci contain four mismatches from the εR136C pegRNA#1 (indicated by lowercase red text). PAM sequences are

shown in green. The Off-target Cutting Frequency Determination (CFD) score for each site is also indicated. (**D**) Sequencing results of εR136C homozygous HEK293T cells at the three predicted most likely off-target sites. No off-target editing was observed at any of the predicted off-target sites.

Supplementary Figure 2

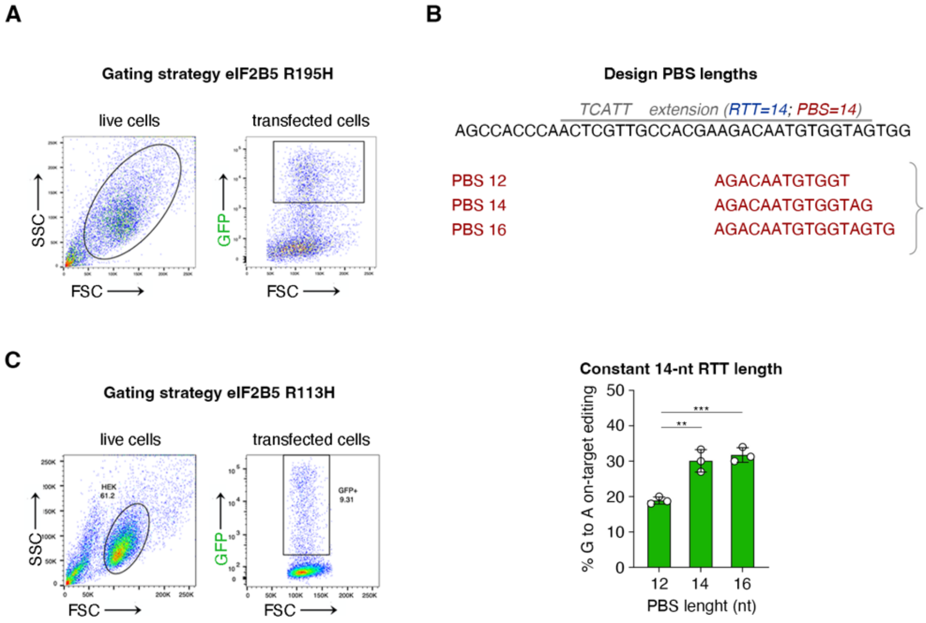

Supplementary Figure 2. PE application to install the εR195H and εR113H mutations in HEK293T cells with optimized pegRNAs. (**A**) Representative FACS plot and gating strategy to sort GFP+ transfected cells using the PE plasmids to install the εR195H mutation in HEK293T cells. (**B**) Top: design of primer binding site (PBS) lengths. Bottom: the corresponding editing efficiencies for G → A substitution generated by the εR195H pegRNA, based on tests of various nucleotide (nt) lengths for the PBS. Dots and bars represent results of three independent transfection experiments and are presented as mean ± sem: **P<0.01; ***P<0.001. (**C**) Representative FACS plot and gating strategy to sort GFP+ transfected cells using the PE plasmids to install the εR113H mutation in HEK293T cells.

Supplementary Figure 3

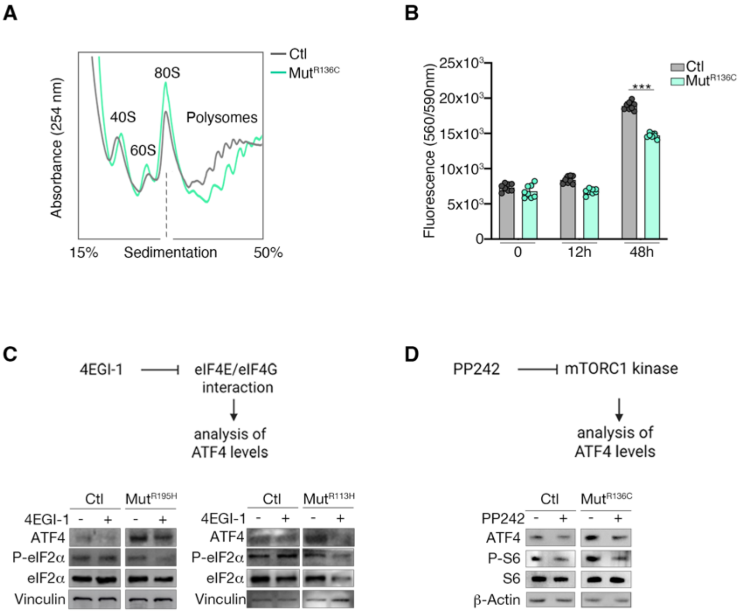

Supplementary Figure 3. Characterization of the activation of the s-ISR in prime-edited HEK293T cells. (**A**) Polysome profile analysis of lysates from Ctl1 and Ctl2 (pooled together) and R136C Mut1 and R136C Mut2 (pooled together); monosomes 40S, 60S, 80S and polysomes are indicated. (**B**) Cell proliferation assay of Ctl and Mut cells performed at the indicated time points. Data are presented as the mean ± s.d. p values are determined by two-tailed Student's t-tests: ***P<0.001. (**C**) Representative immunoblot showing that the eIF4E inhibitor 4EGI-1 disrupts the s-ISR-dependent induction of ATF4 in both in εR195H (left) and εR113H (right) mutants. Vinculin was used as loading control. (**D**) Representative immunoblot from two independent experiments showing the abolishment of ATF4 induction by mTOR inhibitor, which is consistent with eIF4E-dependent ATF4 induction. β-Actin was used as the loading control.

Supplementary Figure 4

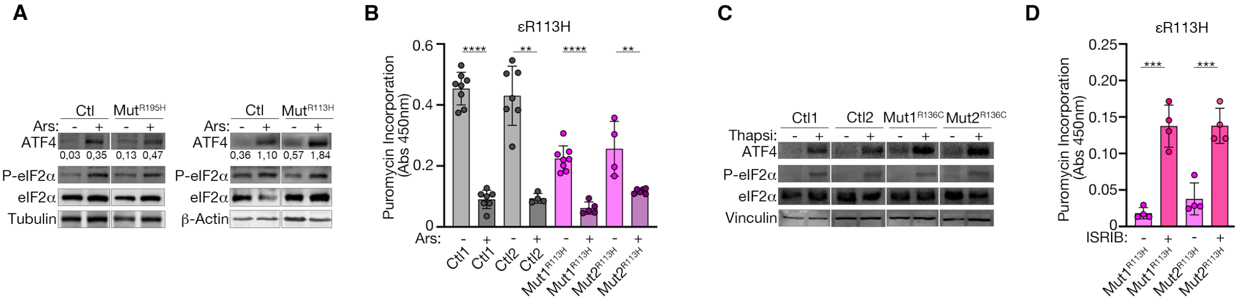

Supplementary Figure 4. Stress worsens the s-ISR in prime-edited mutant HEK293T cells, that can be rescued by ISRIB. (**A**) Representative immunoblots showing ATF4, P-eIF2α and eIF2α levels in εR195H (left) and εR113C (right) mutants, either untreated or treated with arsenite. Tubulin and β-Actin were used as loading controls for the εR195H and εR113C samples, respectively. The numerical values representing the relative levels of ATF4 expression are also indicated. (**B**) Puromycin incorporation in Ctl and εR113H Mut cells left untreated or treated with Arsenite. Data are presented as the mean ± s.d. p values are determined by two-tailed Student's t-tests: **P<0.01; ****P< 0.0001. (**C**) Representative immunoblots showing ATF4, P-eIF2α and eIF2α levels in εR136C mutants, either untreated or treated with Tg. Vinculin was used as loading control. (**D**) Puromycin incorporation in Ctl and εR113H Mut cells left untreated or treated with ISRIB. Data are presented as the mean ± s.d. p values are determined by two-tailed Student's t-tests. ***P< 0.001.

Supplementary Figure 5

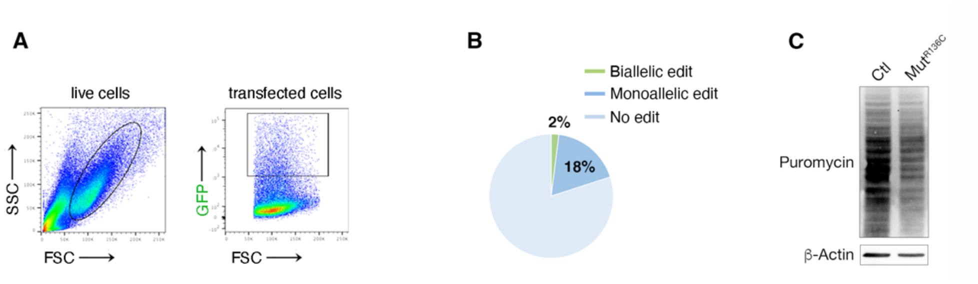

Supplementary Figure 5. Generation of εR136C mutant iPSC lines. (**A**) Representative FACS plot and gating strategy to sort GFP^{+} iPS cells. (**B**) Editing efficiency shown to generate εR136C iPSC by pegRNA#1. (**C**) Immunoblot representative of two independent experiments showing puromycin incorporation in Ctl and Mut iPS. β-Actin was used as loading control.

Supplementary Figure 6

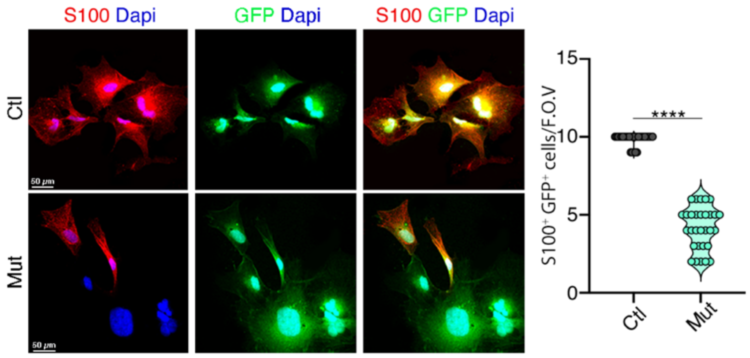

Supplementary Figure 6. εR136C mutation impairs astrocyte maturation. Representative immunofluorescence images of S100B expression (red) in Ctl and Mut iSOX9-astrocytes at 5 days after stopping doxycycline treatment. Dapi was used to stain nuclei. Scale bars, 50 μm. Right, quantification of the percentage of $GFP^{+}S100B^{+}$ cells. Data are shown as the mean ± s.d. p values are determined by two-tailed Student's t-tests: ****$P<0.0001$.

Supplementary Table Legends

Supplementary Table 1. List of sgRNAs candidates.

Supplementary Table 2. Sequence of pegRNAs used in this study. The sequences of pegRNAs are shown in a 5' to 3' orientation.

Supplementary Table 3. Sequence of nicking sgRNAs used in this study.

Supplementary Table 4. Sequences of qRT-PCR sequencing primers used in this study. All sequences are listed in the 5' to 3' orientation.

Supplementary Table 5. Primary antibodies used in this study.
