# Supplementary material for: Prime editing links the split integrated stress response to pathogenic eIF2B mutations and white matter degeneration
Source: Cell Death Dis. 2025 Dec 27;17(1):141. doi: 10.1038/s41419-025-08399-x (PMC12848117; doi:10.1038/s41419-025-08399-x)
Supplement: Supplementary file 2 — Western-blot_full unedited gel [file 41419_2025_8399_MOESM2_ESM.pdf]

Figure 2_D_R136C

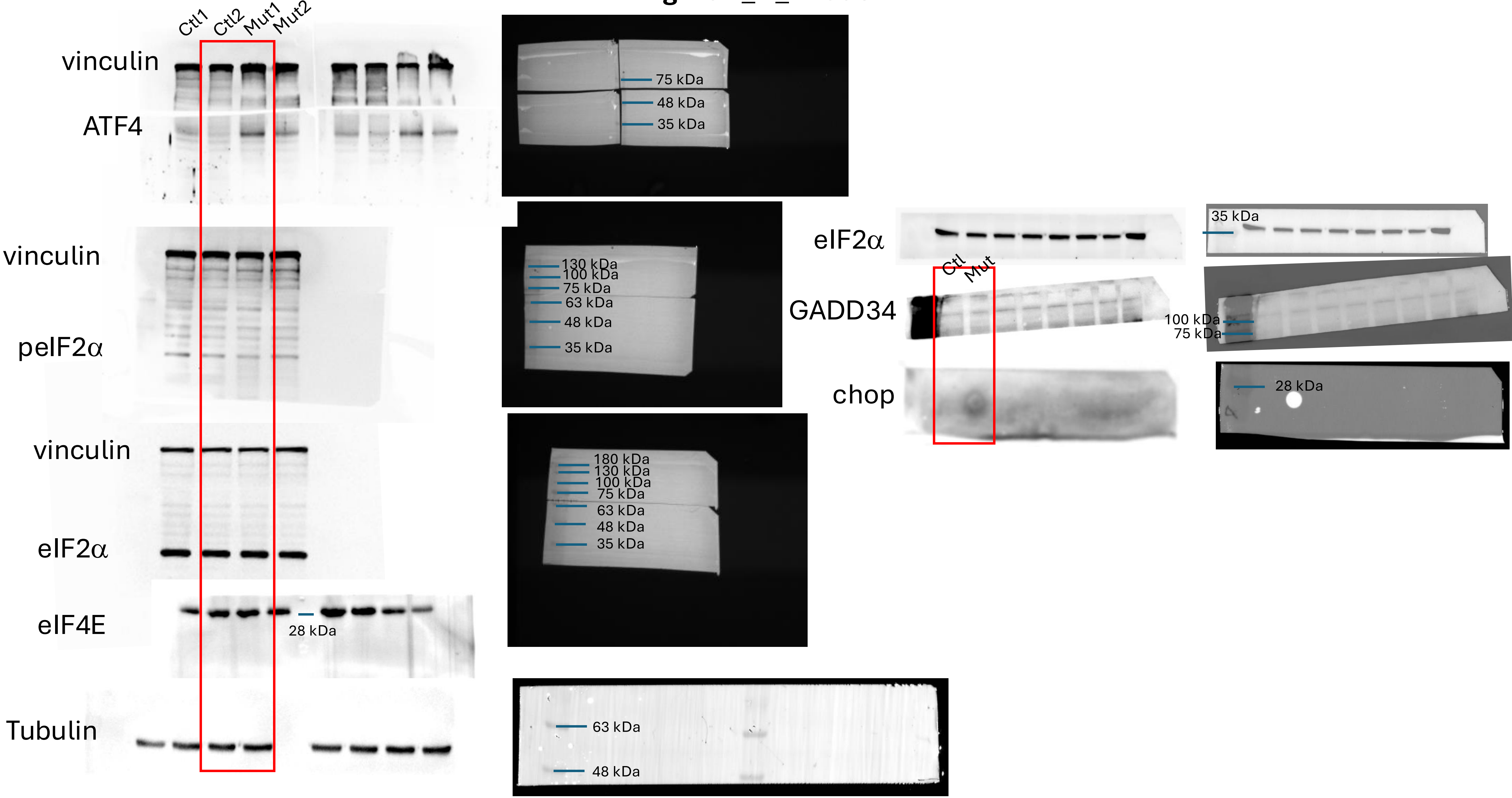

Figure 2_D_R195H

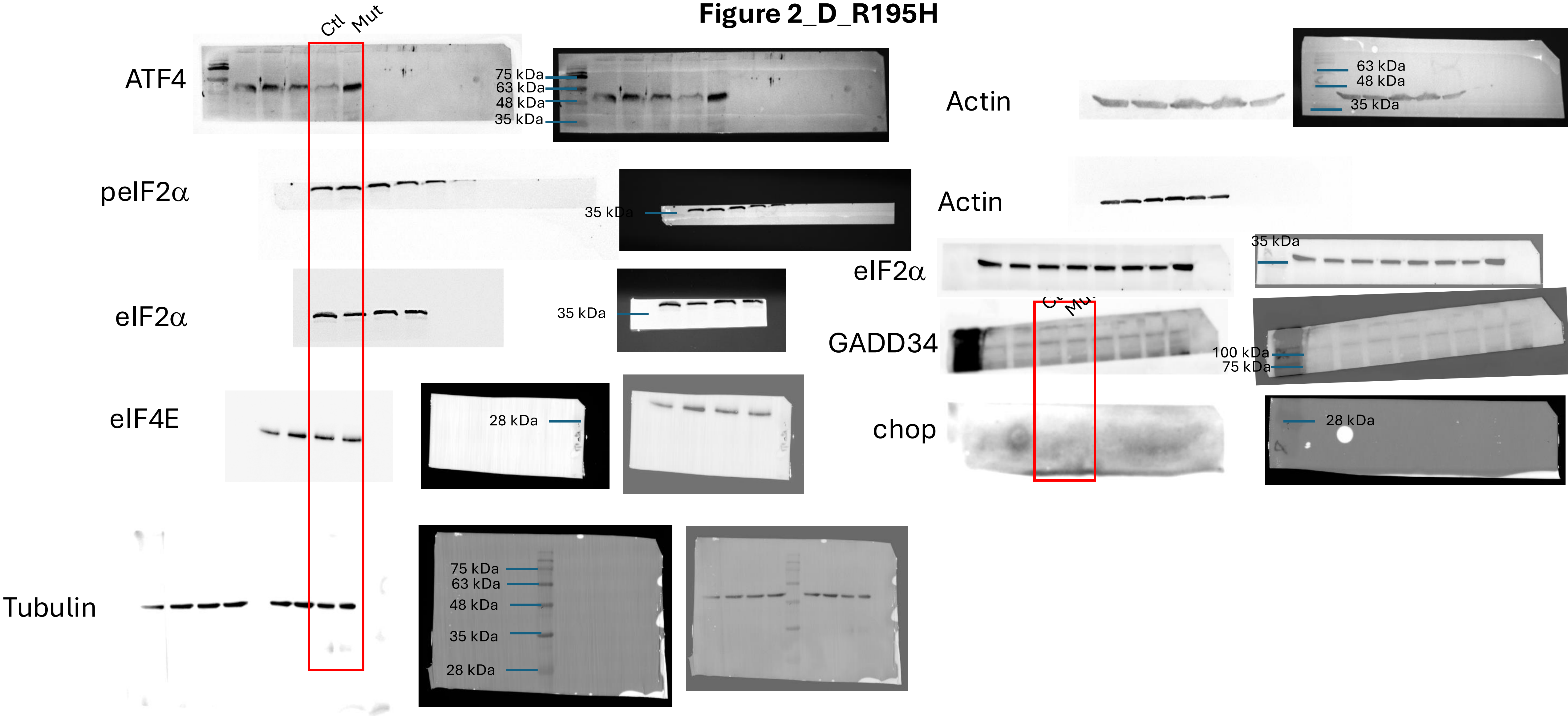

Figure 2_D_R113H

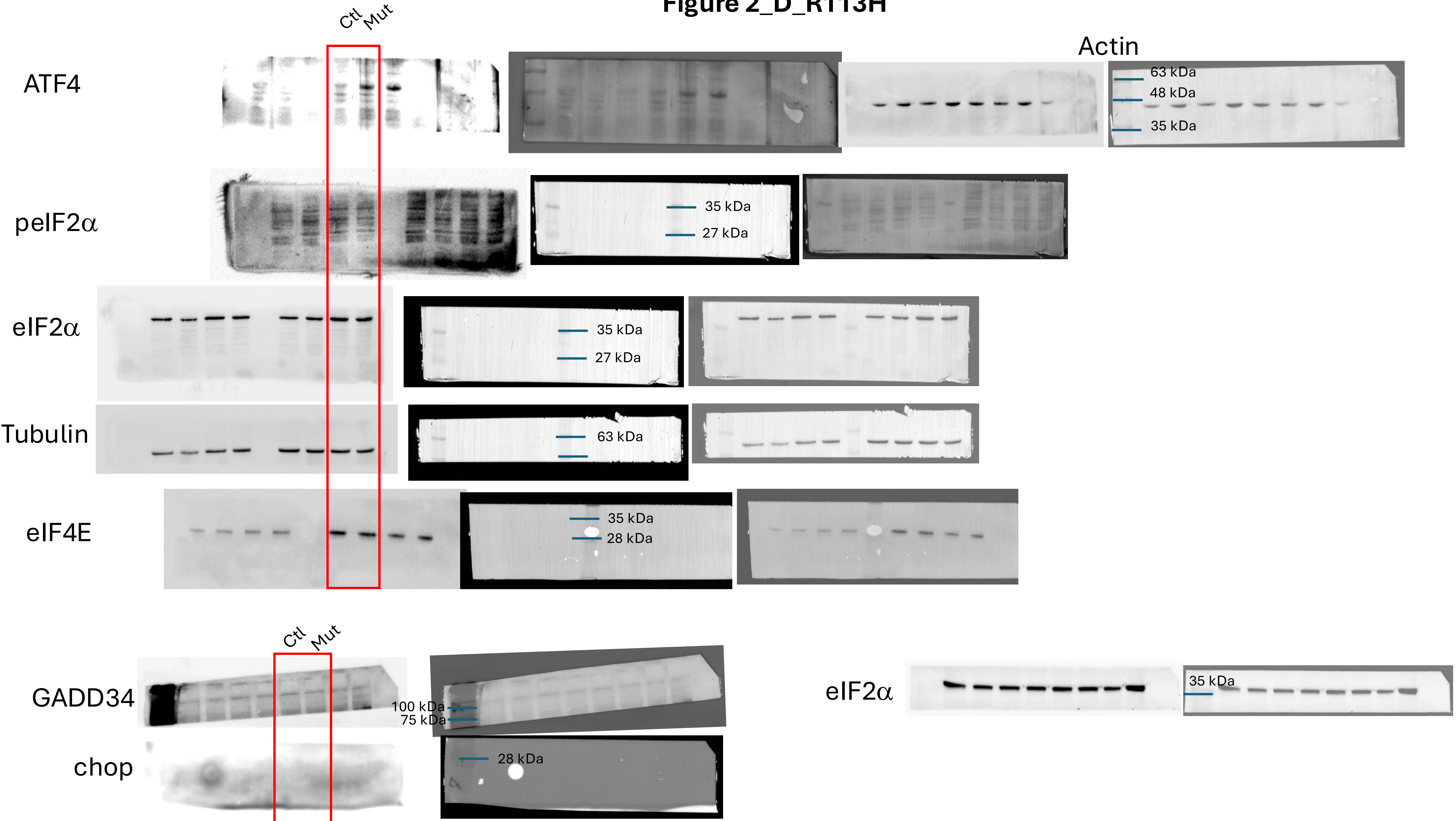

Figure 2_G

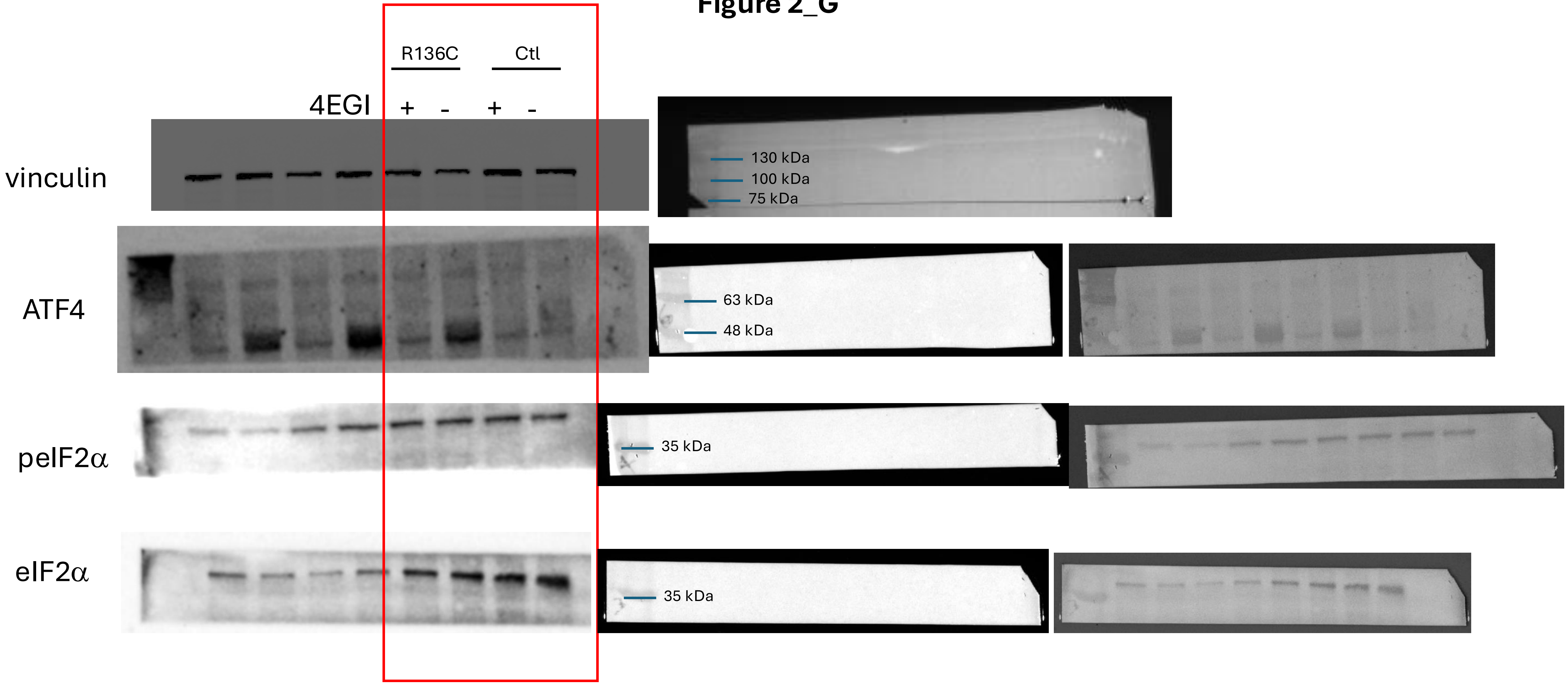
R136C
Ctl
4EGI
+
-
+
-
vinculin
ATF4
peIF2α
eIF2α
130 kDa
100 kDa
75 kDa
63 kDa
48 kDa
35 kDa
35 kDa

Figure 3_A

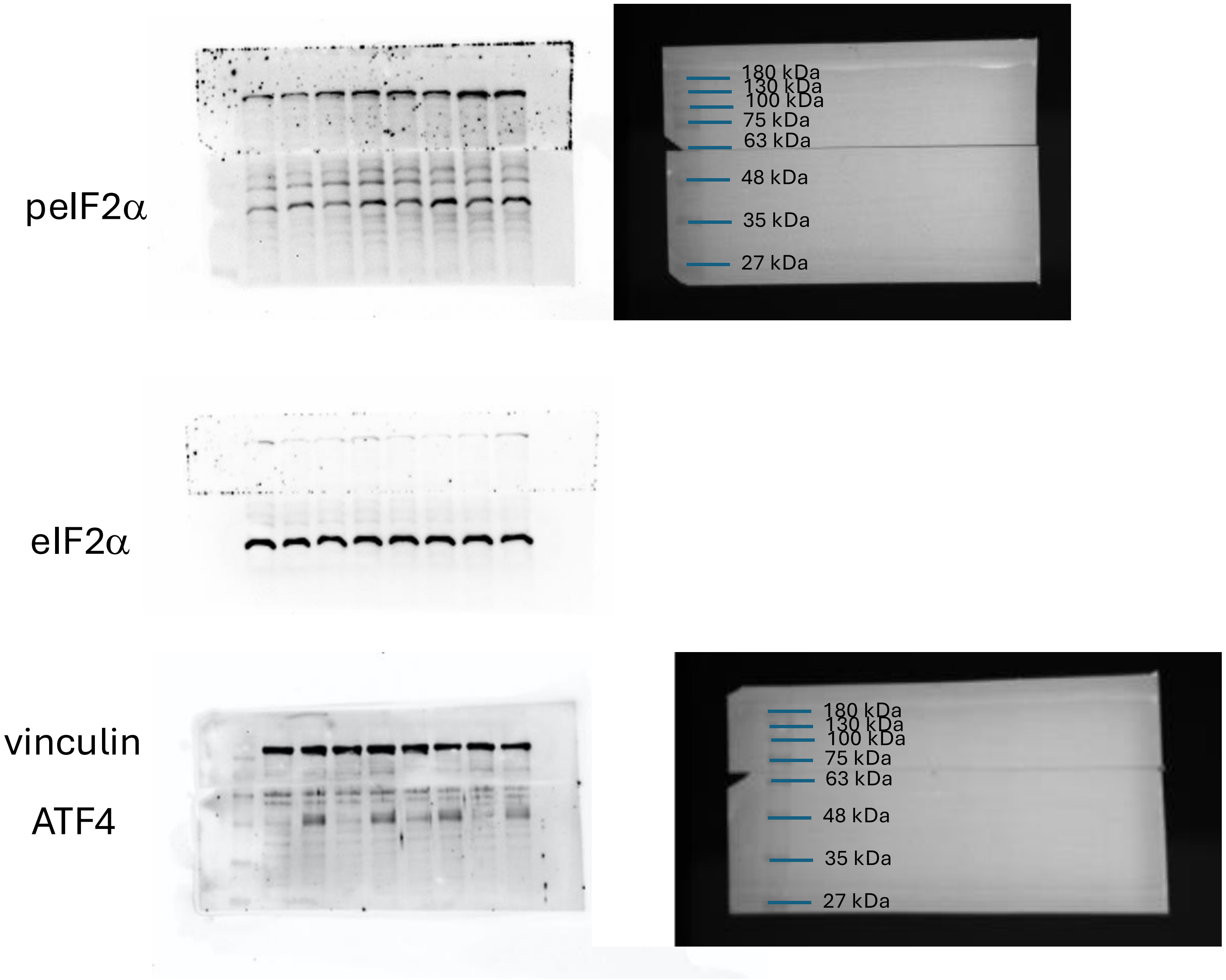

peIF2α
180 kDa
130 kDa
100 kDa
75 kDa
63 kDa
48 kDa
35 kDa
27 kDa
eIF2α
vinculin
ATF4
180 kDa
130 kDa
100 kDa
75 kDa
63 kDa
48 kDa
35 kDa
27 kDa

Figure 3_C

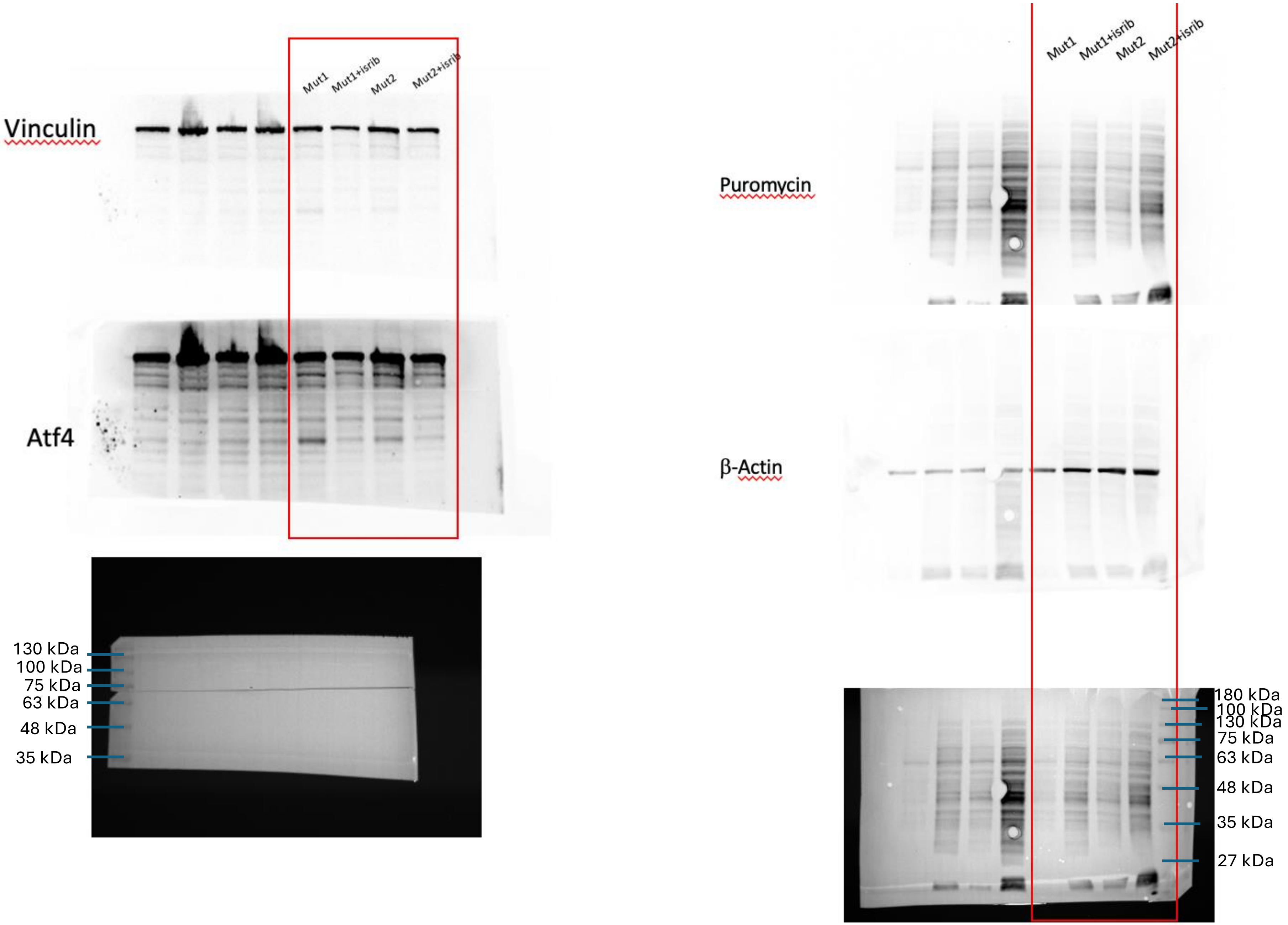
Mut1
Mut1+isrib
Mut2
Mut2+isrib
Vinculin
Atf4
130 kDa
100 kDa
75 kDa
63 kDa
48 kDa
35 kDa
Puromycin
β-Actin
180 kDa
100 kDa
130 kDa
75 kDa
63 kDa
48 kDa
35 kDa
27 kDa

Figure 3_E

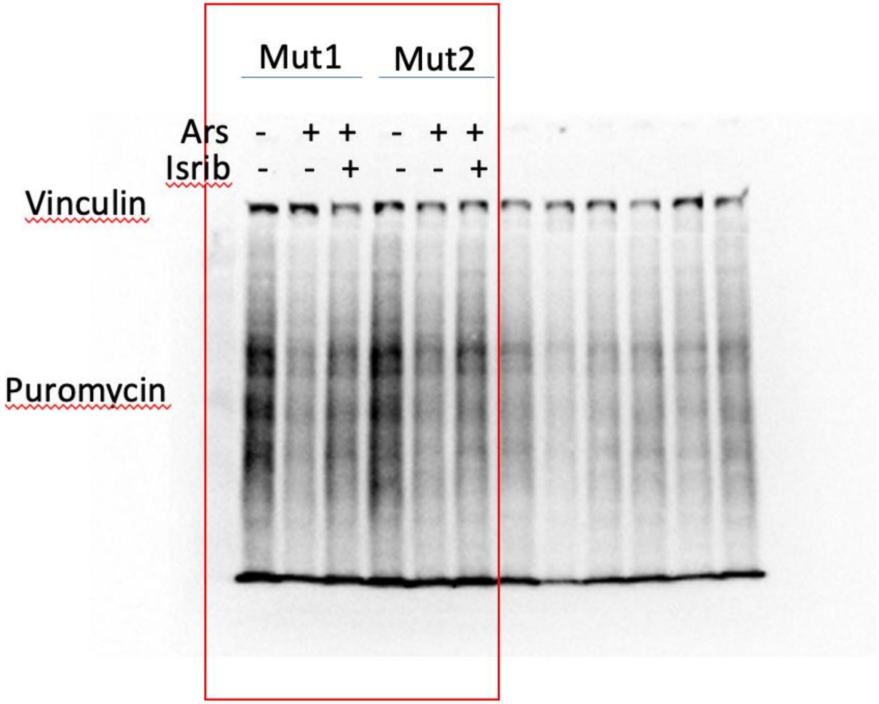
Mut1
Mut2
Ars - + + - + +
Isrib - - + - - +
Vinculin
Puromycin

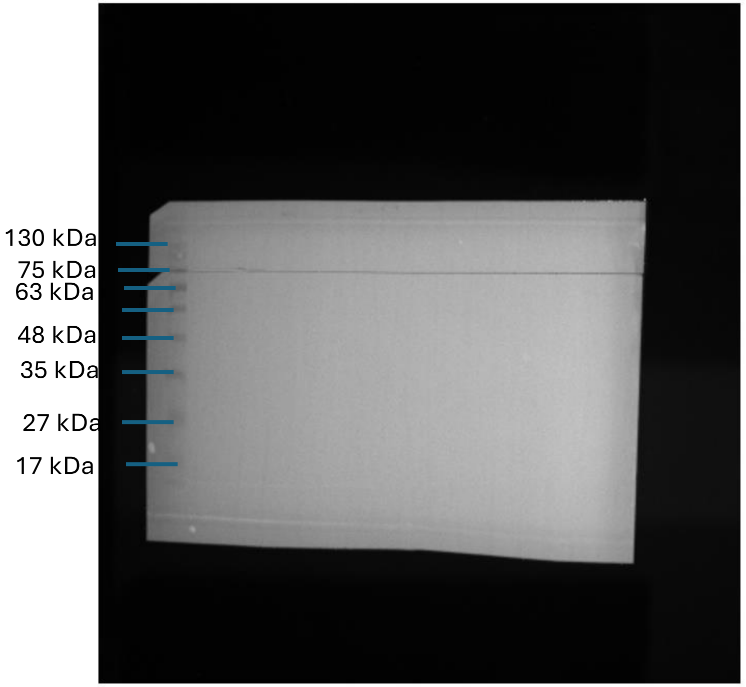
130 kDa
75 kDa
63 kDa
48 kDa
35 kDa
27 kDa
17 kDa

Figure 4_E

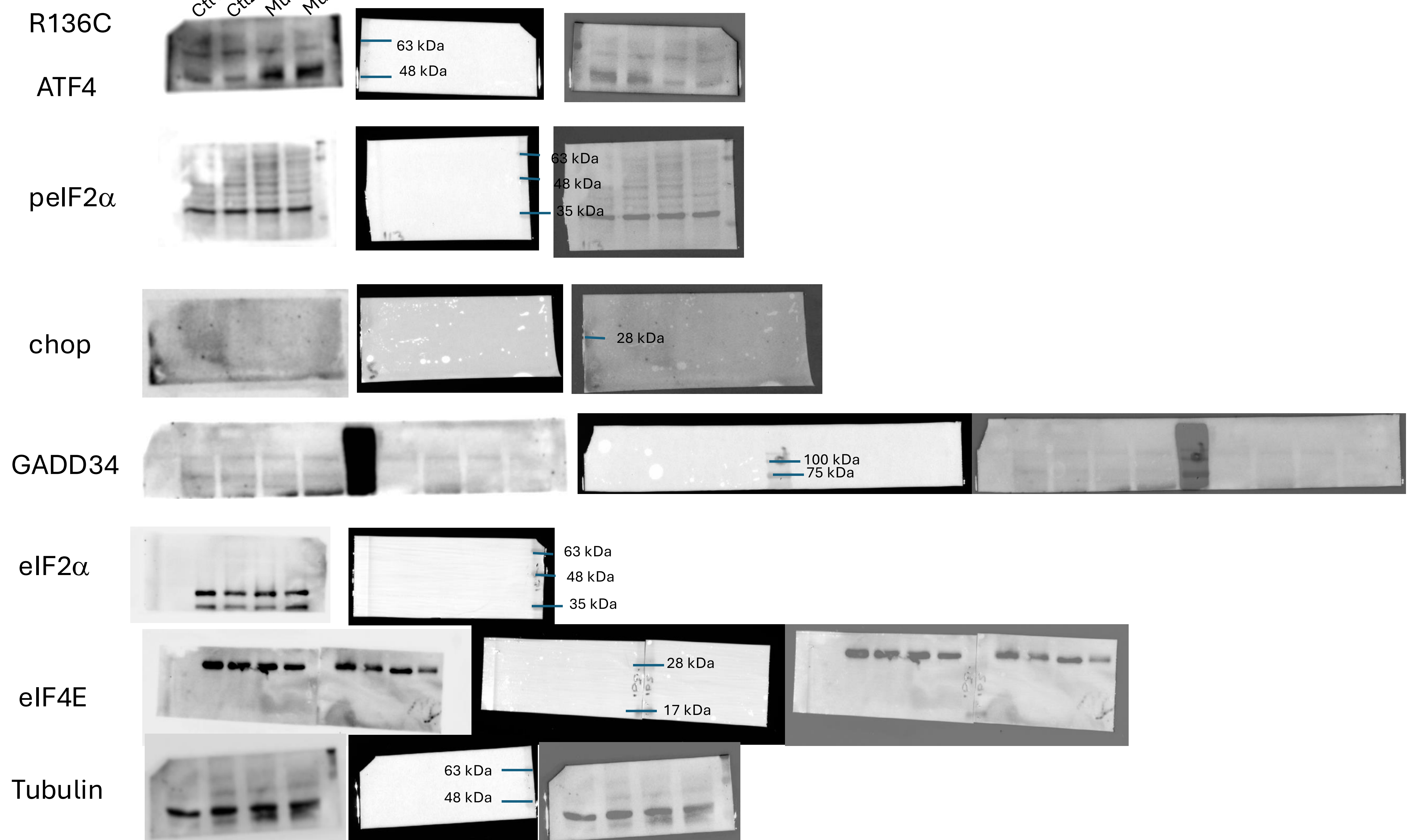

Ctl1
Ctl2
Mut1
Mut2
R136C
ATF4
63 kDa
48 kDa
peIF2α
63 kDa
48 kDa
35 kDa
chop
28 kDa
GADD34
100 kDa
75 kDa
eIF2α
63 kDa
48 kDa
35 kDa
eIF4E
28 kDa
17 kDa
Tubulin
63 kDa
48 kDa

Figure 4_F

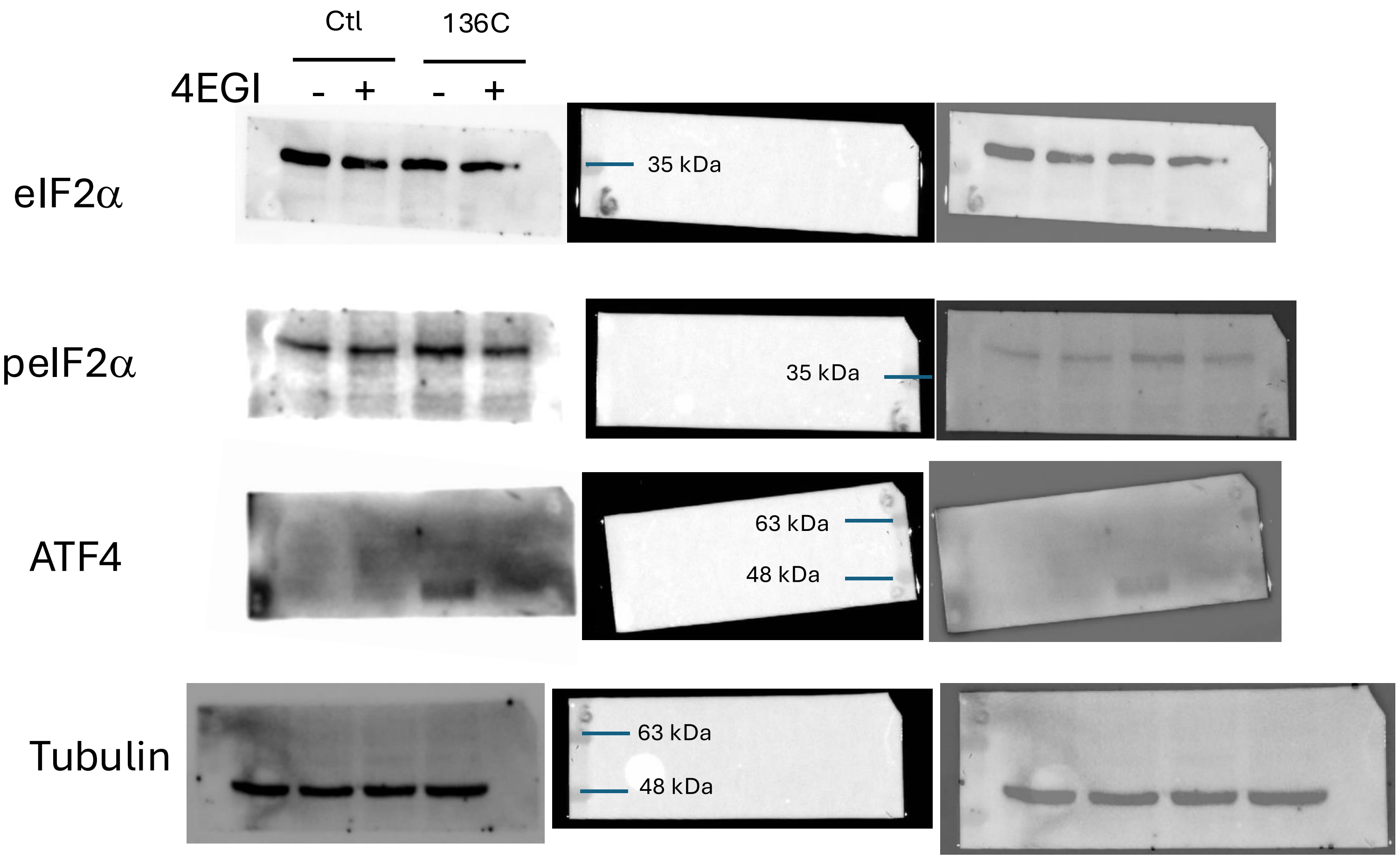

Ctl
136C
4EGI - + - +
eIF2α
35 kDa
peIF2α
35 kDa
ATF4
63 kDa
48 kDa
Tubulin
63 kDa
48 kDa

Figure 4_H

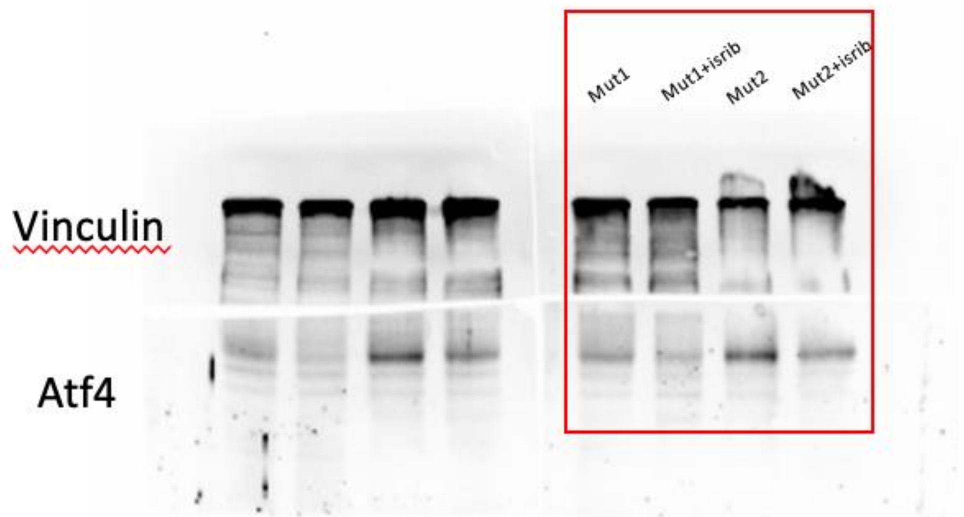
Mut1
Mut1+isrib
Mut2
Mut2+isrib
Vinculin
Atf4

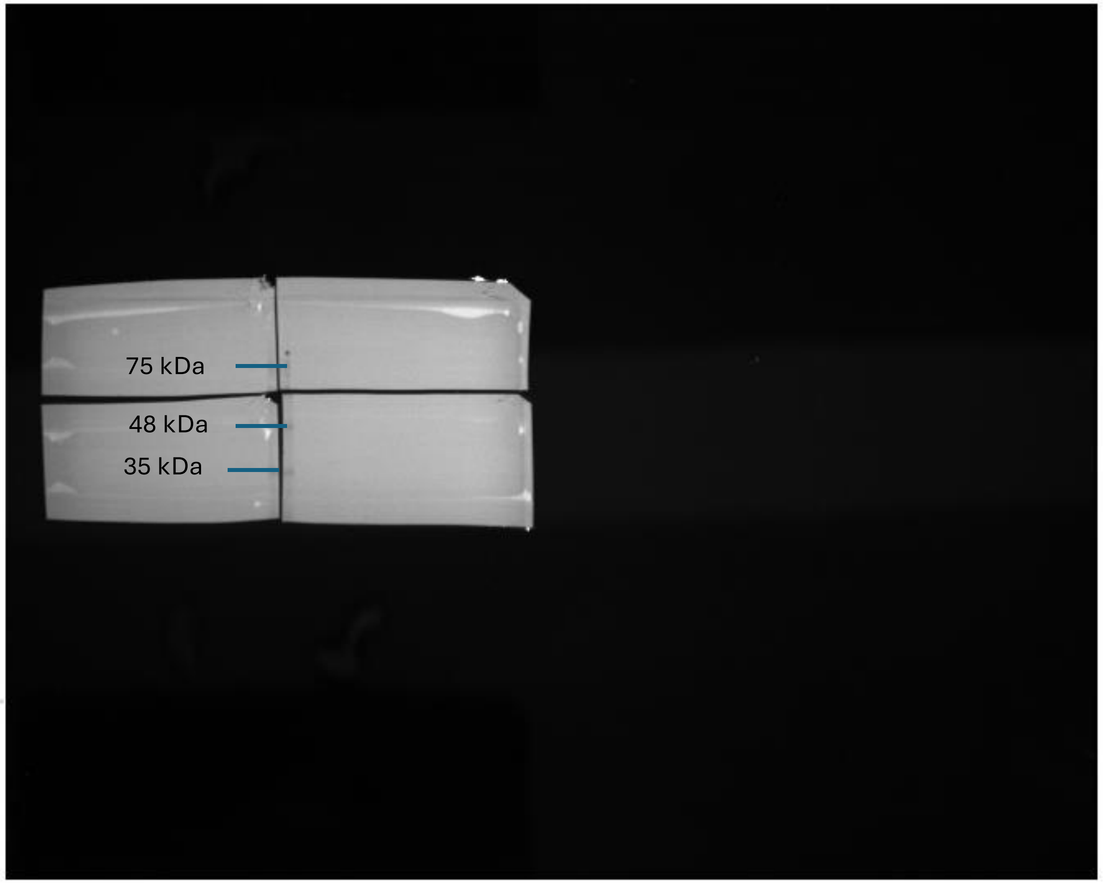
75 kDa
48 kDa
35 kDa

Figure S3_C_left

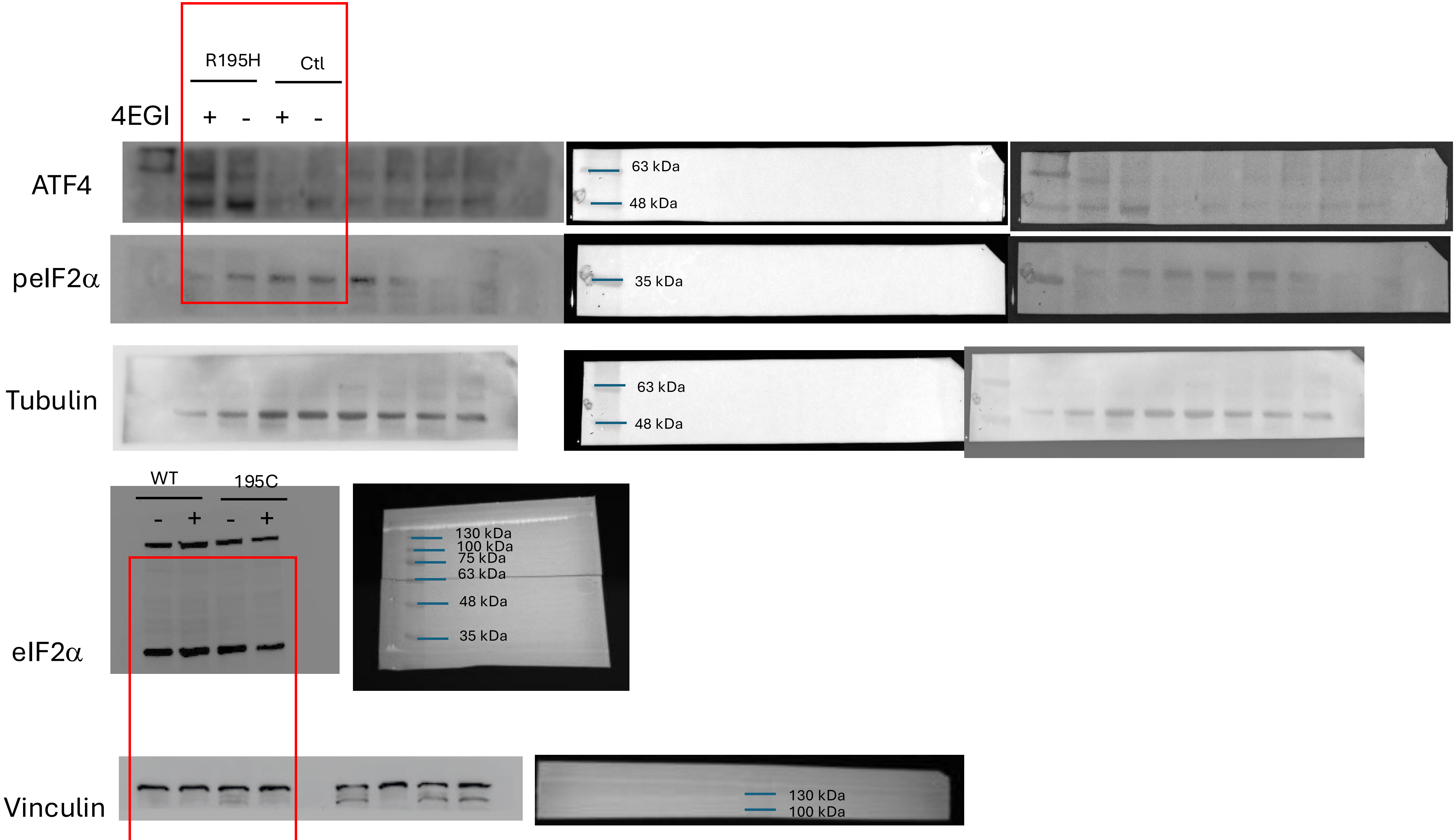

Figure S3_C_right

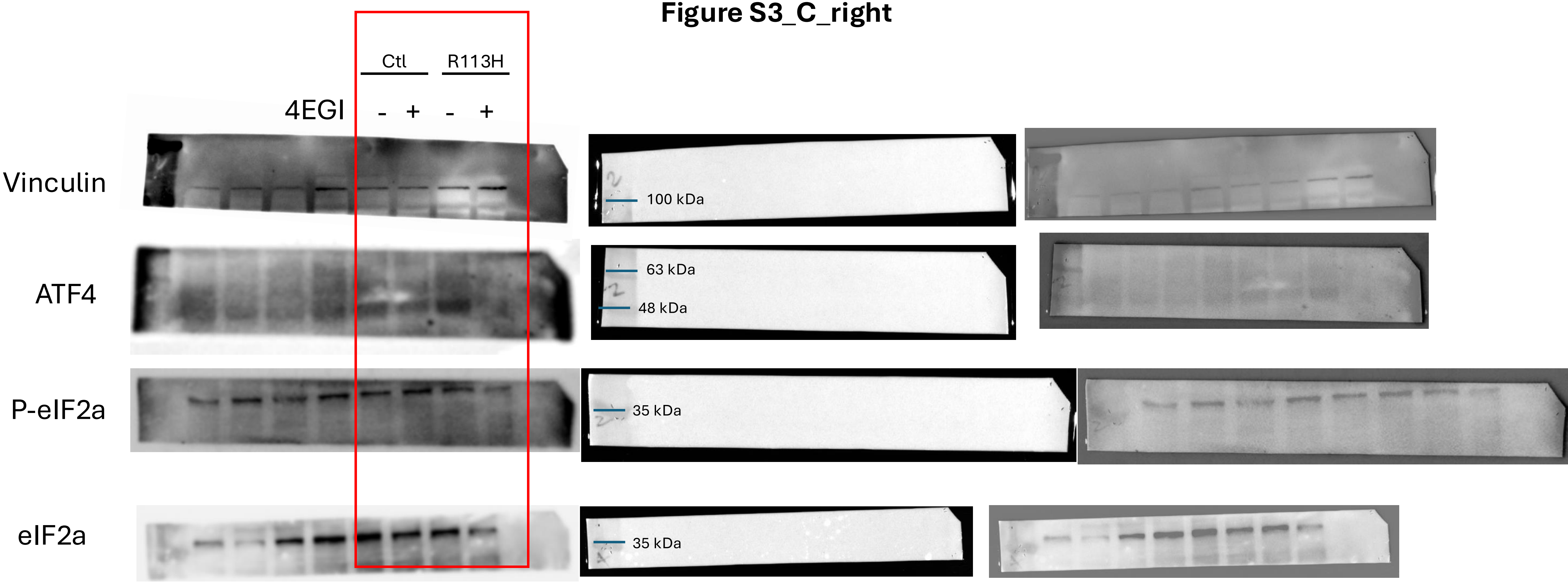

Figure S3_D

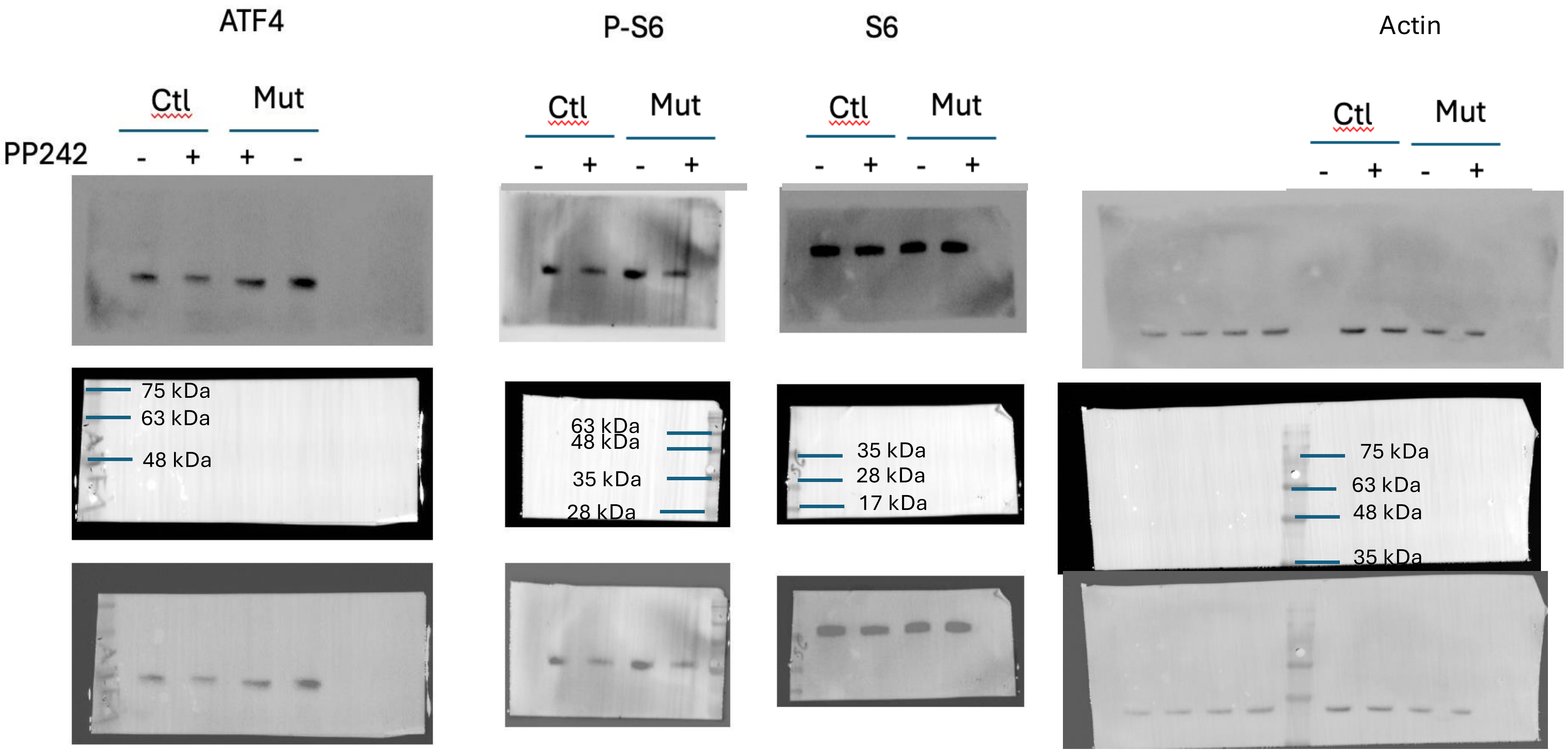

ATF4
P-S6
S6
Actin
Ctl
Mut
PP242
- + + -
- + - +
75 kDa
63 kDa
48 kDa
35 kDa
28 kDa
17 kDa

Figure S4_A_left (R195H)

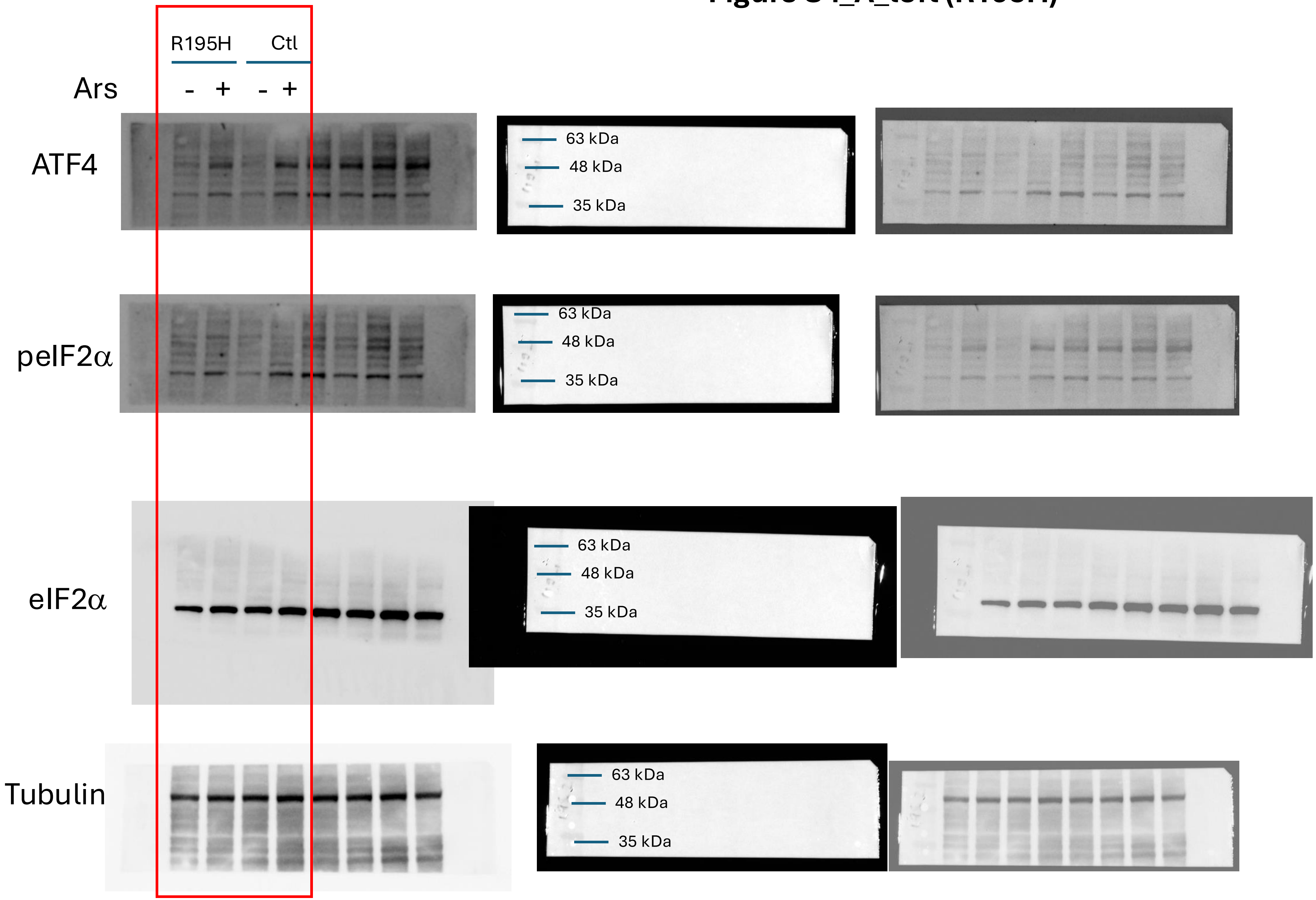

Figure S4_A_right (R113H)

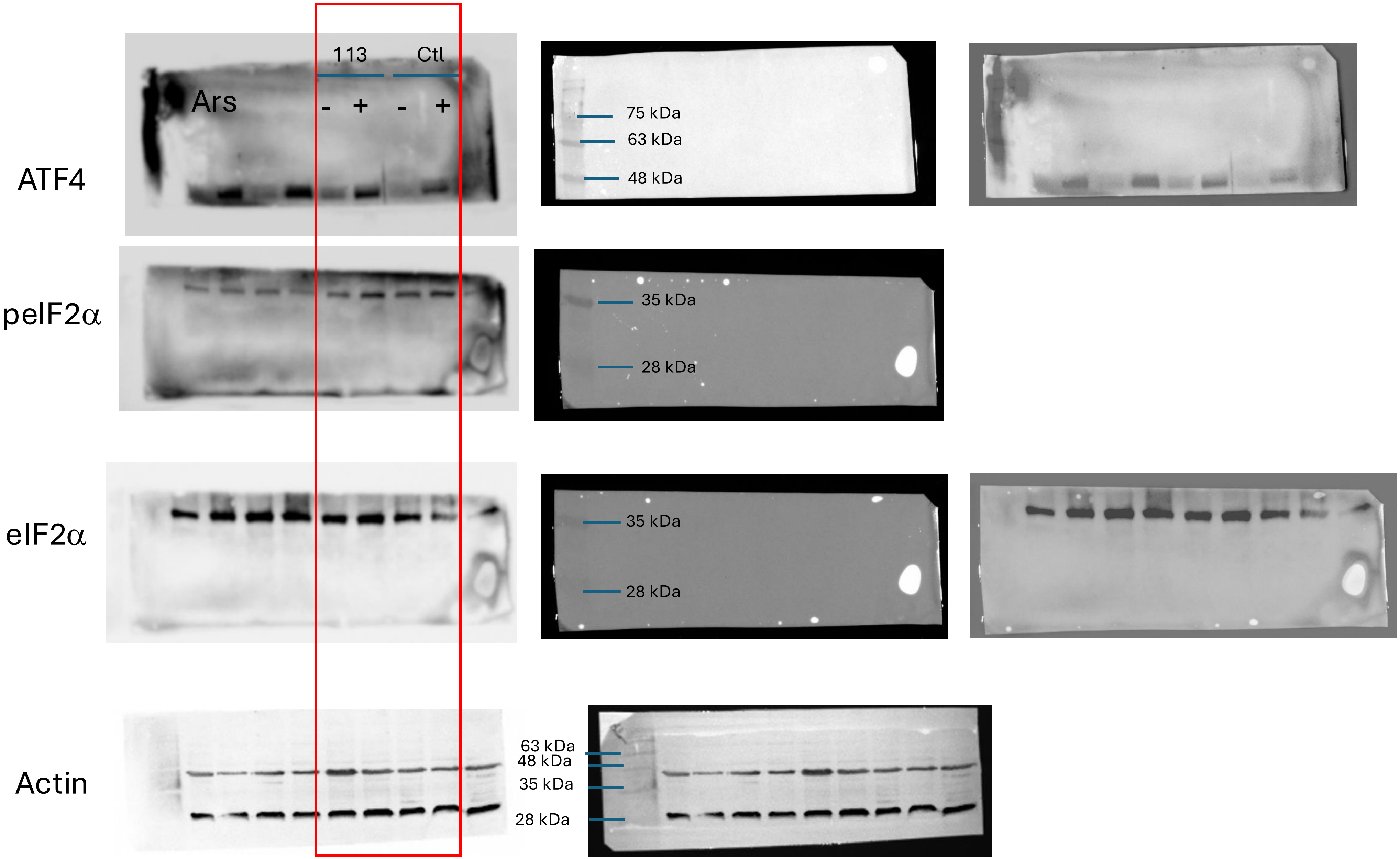

Figure S4_C

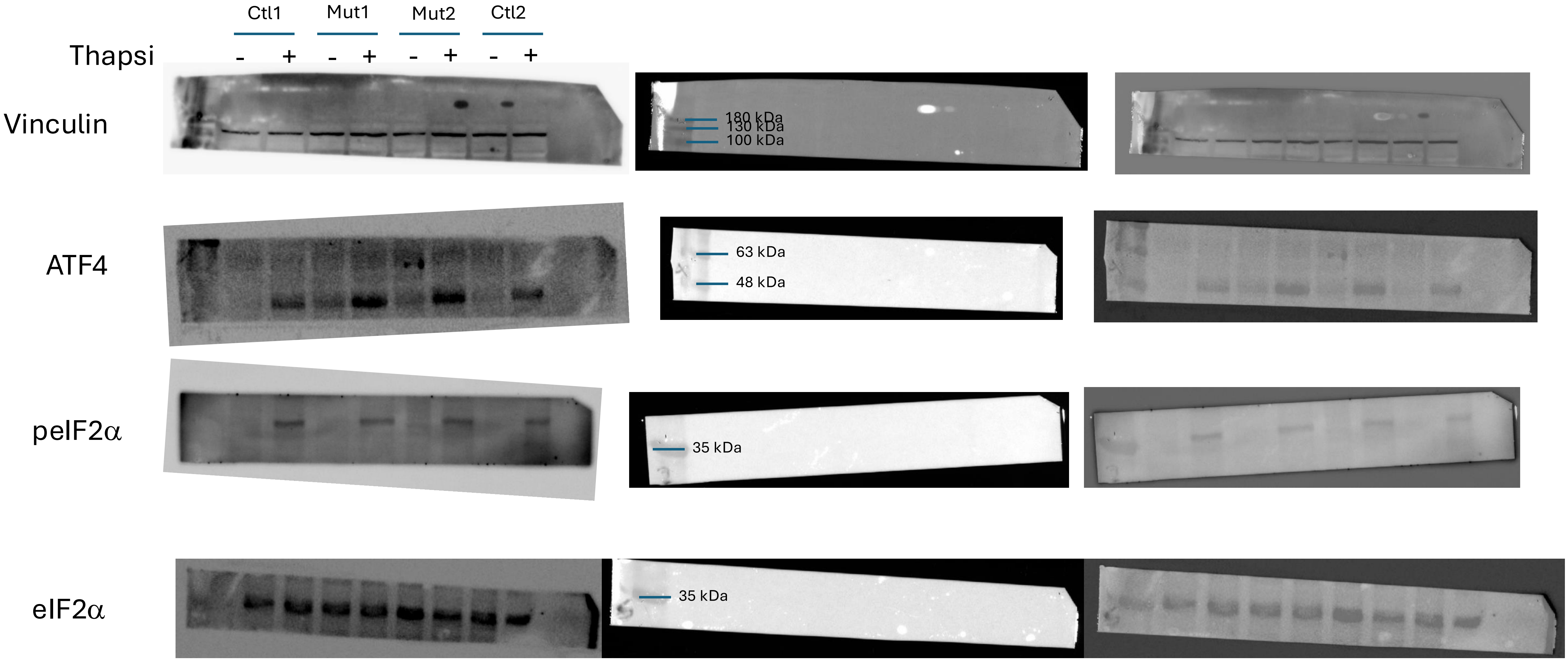

Ctl1
Mut1
Mut2
Ctl2
Thapsi
- + - + - + - +
Vinculin
180 kDa
130 kDa
100 kDa
ATF4
63 kDa
48 kDa
peIF2α
35 kDa
eIF2α
35 kDa

Figure S5_C

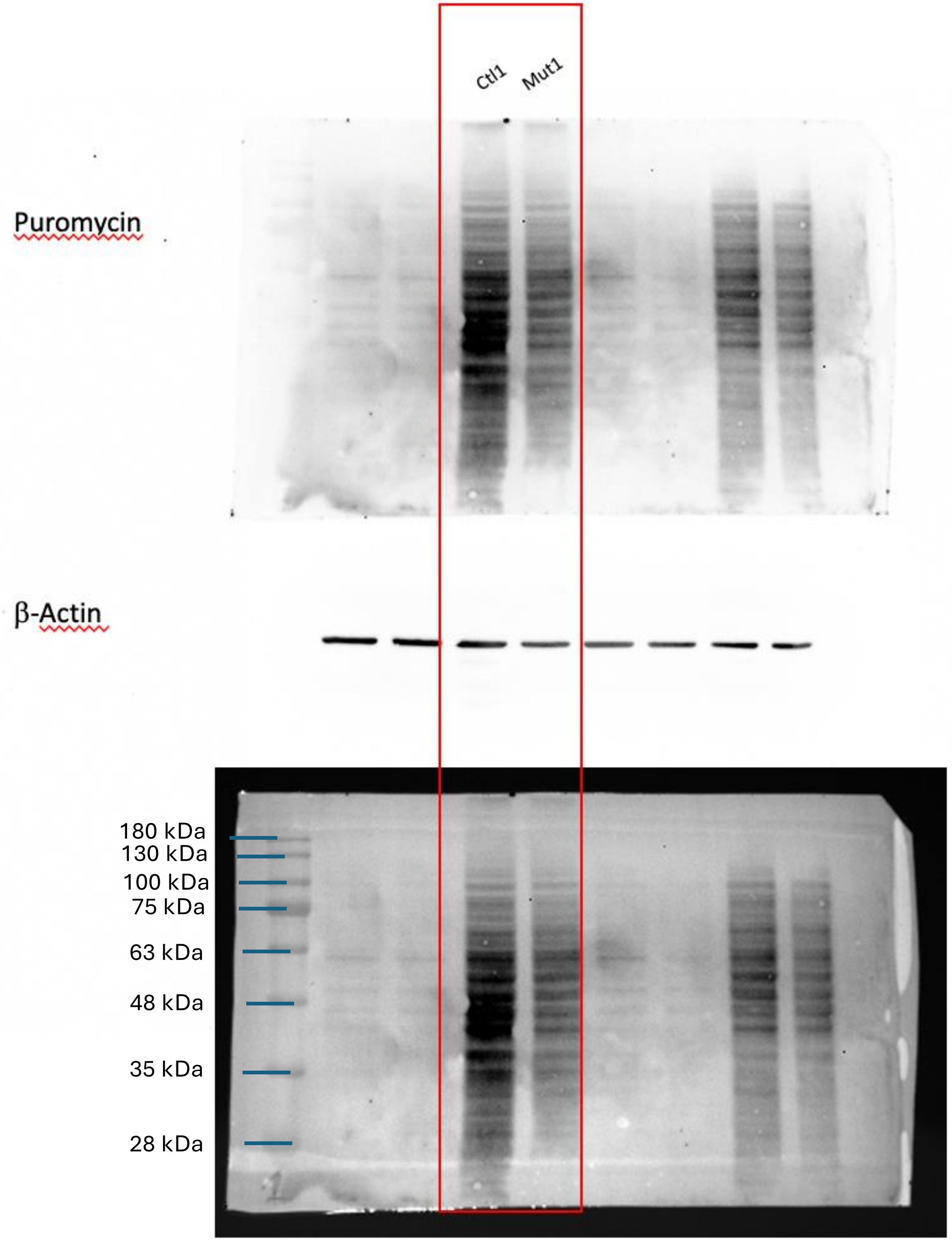
Ctl1
Mut1
Puromycin
β-Actin
180 kDa
130 kDa
100 kDa
75 kDa
63 kDa
48 kDa
35 kDa
28 kDa
